# Dimensions of control for subthreshold oscillations and spontaneous firing in dopamine neurons

**DOI:** 10.1371/journal.pcbi.1007375

**Published:** 2019-09-23

**Authors:** Timothy Rumbell, James Kozloski

**Affiliations:** IBM Research, Computational Biology Center, Thomas J. Watson Research Laboratories, Yorktown Heights, New York, United States of America; Ernst-Strungmann-Institut, GERMANY

## Abstract

Dopaminergic neurons (DAs) of the rodent *substantia nigra pars compacta* (SNc) display varied electrophysiological properties *in vitro*. Despite this, projection patterns and functional inputs from DAs to other structures are conserved, so *in vivo* delivery of consistent, well-timed dopamine modulation to downstream circuits must be coordinated. Here we show robust coordination by linear parameter controllers, discovered through powerful mathematical analyses of data and models, and from which consistent control of DA subthreshold oscillations (STOs) and spontaneous firing emerges. These units of control represent coordinated intracellular variables, sufficient to regulate complex cellular properties with radical simplicity. Using an evolutionary algorithm and dimensionality reduction, we discovered metaparameters, which when regressed against STO features, revealed a 2-dimensional control plane for the neuron’s 22-dimensional parameter space that fully maps the natural range of DA subthreshold electrophysiology. This plane provided a basis for spiking currents to reproduce a large range of the naturally occurring spontaneous firing characteristics of SNc DAs. From it we easily produced a unique population of models, derived using unbiased parameter search, that show good generalization to channel blockade and compensatory intracellular mechanisms. From this population of models, we then discovered low-dimensional controllers for regulating spontaneous firing properties, and gain insight into how currents active in different voltage regimes interact to produce the emergent activity of SNc DAs. Our methods therefore reveal simple regulators of neuronal function lurking in the complexity of combined ion channel dynamics.

## Author summary

Electrophysiological activity of the neuronal membrane and concomitant ion channel properties are highly variable within groups of neurons of the same type from the same brain region. Reconciliation of the mechanisms generating neuronal activity is challenging due to the complexity of the interactions between the channel currents involved. Here we present a set of mathematical analyses that uncover the low-dimensional intracellular parameter combinations capable of regulating features of subthreshold oscillations and spontaneous firing in empirically constrained models of nigral dopaminergic neurons. This method generates, from a naive starting point, linear combinations of ion channel properties that are surprisingly capable of reliably controlling a wide variety of emergent electrophysiological activity, thereby predicting drug effects and shedding light on unsuspected compensatory mechanisms that contribute to neuronal function.

## Introduction

Midbrain dopaminergic neuron (DA) activity influences many brain functions. Timing of spikes and bursts in DAs signal reward prediction error [1], and DA activity is implicated in schizophrenia and depression [2]. Modeling the origins of DA activity is vital for research into the neural basis of behavior and neuropsychiatric therapeutic design.

Correlations exist between DA ion channel properties and electrophysiology. A-type potassium (KA) channels regulate spike timing, with negative correlations between current amplitude and interspike interval voltage slope [3], and between the inactivation time constant and rebound voltage slope [4]. Hyperpolarization-activated (HCN) channel density regulates rebound delay [5]. Modulating calcium-dependent potassium (SK) channels can alter firing rate (FR) [6], FR adaptation ratio [7], or modulate firing from regular spiking to bursting [8]. KA density modulates spontaneous FR [9] as does blockade of ERG potassium [10]. Blockade of T-type calcium modulates rebound spiking [11]. Subthreshold membrane potential dynamics ultimately help determine the impact of a presynaptic spike and whether DAs fire, making the interplay between these channels crucial for controlling DAs and their role in brain function. Analysis of populations of electrophysiological models has previously been used to uncover the influence of ion channel parameters on output features [12, 13]. Here, we generate and analyze a population of DA models, discovering powerful, simultaneous controllers of multiple functions, and thereby providing a means to formulate quantitative hypotheses about polychannel regulatory targets in the real neurons.

Characterization of subthreshold activity in *substantia nigra pars compacta* (SNc) DAs has elucidated how channels activated in the subthreshold regime combine to regulate responses. Here, a subthreshold oscillation (STO) emerges in the presence of tetrodotoxin (TTX), with increased amplitude under tetraethylammonium (TEA) [14, 15]. STOs depend on L-type calcium activation [16] resulting in depolarization terminated by SK. The characteristics of these STOs reveal the balance of subthreshold currents present. Prior work had hypothesized that the spontaneous pacemaker firing observed in DAs *in vitro* was entrained by the STOs [17], but recent evidence has indicated that persistent sodium current is a likely driver of pacemaker firing [18, 19], rather than STO frequency. Computational models of DAs have successfully reproduced STOs and elucidated their dependence on interactions between calcium and SK [17, 20], but no previous studies reproduced the full range of STOs or their functional regulation over this range. Furthermore, prior computational models that may provide insight into the interactions between DA activity modes have used single parameter sets producing single activity patterns [4, 20, 21], whereas a range of spontaneous oscillation and spiking features have been observed. We wondered if a parsimonious means to modulate STOs over their full range was discoverable in a population of models.

First we reasoned that electrophysiological measures from neuronal populations reveal feature variability across cells of the same type [22, 23]. Varied features derive from varied ion channel expression, current densities [9], kinetic gating [24, 25], and morphology [26–28]. Differences in these properties somehow balance within each cell, resulting in similar function for neurons with different underlying properties [29–31]. Pharmacological manipulations across cells of the same type can have variable effects, seen in the variable responses of DAs to apamin [32], but how variability observed in ion channel properties contributes to variability in electrophysiological properties has yet to be fully characterized [33].

Here we apply a novel method for population parameter search and dimensionality reduction to address these issues. Our analysis of a population of algorithmically generated DA models produces low-dimensional mappings of ion channel properties onto STOs. Our technique identifies a region of model parameter space in which linear operators vary STO features and regenerate the complete DA population activity by coregulating ion channels and controlling the models’ emergent electrophysiology. From this subthreshold-regulating plane, we produce a population of models with the full range of spontaneous firing characteristics and further show how discovery of this population enables identification of the ion channel coregulations that promote variety of DA function. We demonstrate how this model population generalizes to drug perturbations, contains known compensatory mechanisms, and reveals intracellular properties responsible for susceptibility to activity mode state transitions. Finally, we demonstrate a complex relationship between subthreshold and spiking modes, thus providing theoretical insight into the various interpretations of numerous empirical results by showing how the membrane potential history constrains phase shift and frequency modulation of pacemaking, indicating distinct ion channel state profiles during either STO or spontaneous firing activity.

## Materials and methods

### Neuron model

The neuron model used a multi-compartment dendritic morphology based on a previously published DA model morphology [21], with an axon initial segment (AIS) and axonal compartment added. Into this, we incorporated a set of equations determining membrane potential, intracellular calcium, and ion channel gating. The included channels were transient sodium (NaT), hyperpolarization-activated cation (HCN), T-type calcium, Cav3 (CaT), low-threshold L-type calcium, Cav1.3 (CaL), delayed rectifier potassium (Kv2), large-conductance potassium (BK), small-conductance, calcium-dependent potassium (SK), transient A-type potassium, Kv4.3 (KA), and ether-a-go-go-related-gene potassium (KERG), along with the leak conductance. A full description of the channel models used and tuning process applied to those channels to improve their fit to SNc DA observations is provided in **Ion channel tuning procedures**, below. Briefly, for each ion channel we found an appropriate existing model that captured fundamental properties of the channel, adjusted baseline kinetic parameters to approximate available observations from recordings of that channel type in SNc DAs, then inserted parameters into those equations for coherently modulating time constants and voltage dependence within the ranges found in those empirical observations. For the optimization and for each channel model as required, we incorporated parameters $\bar{g}$, maximal conductance, V_half_ for modifying voltage-dependence of the default channel models, *τ*_mod_ for modifying time constants for activation and inactivation, *e*, the reversal potential, and *k*, the Ca^2+^-dependence of a channel gating variable, along with intracellular calcium parameters P_max_ and *β* (see **Membrane potential equation**, below). Additional scaling factor parameters were incorporated to allow the optimization to adjust NaT and Kv2 conductances and kinetics in the AIS.

#### Morphological model

The model consisted of 15 cylindrical compartments, comprising a soma, 4 proximal dendrites, an AIS and a proximal axon. The SNc DA population has a mean of 4 dendritic arbors [26]. Each proximal dendrite in the model branched into 2 distal dendrites. We scaled the length and diameter of each compartment so that somatic surface area, total dendritic length, and total somatodendritic surface area approximated mean experimental observations [26]. Our scaling resulted in soma, proximal and distal dendrite lengths of 18.6, 800, and 400 μm, mean diameters of 18.6, 1.25 and 0.75 μm, and a somatodendritic surface area of 15871 μm^2^. We used a multiple compartment model of soma and dendrites for our study of how STO derives from biophysical ion channel models because of the observations of [17], which showed that calcium dynamics derived from identical channels function differently in soma and dendrites because narrower diameters enhance the rate of change of calcium concentration. The AIS segment emerged from one proximal dendritic section, 30 μm from the soma, with length 30 μm and diameter 1 μm, before continuing as a proximal axonal segment with length 470 μm and diameter 1 μm, approximating the AIS and axon configuration established in [27].

#### Membrane potential equation

The membrane potential *V* of each compartment *k* was updated according to the discretized cable equation:

$$\begin{array}{c} C_{{\text{m}}_{k}}\frac{\delta V_{k}}{\delta t}=\Sigma_{j}g_{{\text{a}}_{j,k}}\left(V_{j}-V_{k}\right)-I_{\text{ionic},k}-I_{\text{Leak}}, \end{array}$$ (1)

where the membrane capacitance $C_{{\text{m}}_{k}}=C_{\text{m}}\pi d_{k}l_{k}$ (with specific membrane capacitance *C*_m_, compartment length *l*_*k*_, and diameter *d*_*k*_). The sum Σ_*j*_ represents the total axial current from compartment *k* to all adjacent compartments *j*, computed as the voltage difference (*V*_*j*_ − *V*_*k*_) times the axial conductance $g_{{\text{a}}_{j,k}}=\frac{4R_{\text{a}}l_{k}}{\pi d_{k}^{2}}$ (with specific axial resistivity *R*_a_). *C*_m_ was 0.75 μF/cm^2^ and *R*_a_ was 100 Ω cm, according to [27].

For each ionic current model (see below), *I*_ionic,*k*_ was calculated as

$$\begin{array}{c} I_{\text{ionic},k}=g_{\text{ionic},k}\left(V_{k}-E_{\text{ionic}}\right), \end{array}$$ (2)

where *g*_ionic,*k*_ is channel conductance in compartment *k*, and *E*_ionic_ is reversal potential for the relevant ionic species. Channel conductances were identical throughout all soma and dendritic compartments. Spatial inhomogeneity was not considered, although greater current densities at the soma have been demonstrated for KA [34] and at the dendritically-located axon initial site for HCN [35]. We did not include these inhomogeneities because of incomplete data surrounding other channel types, and the additional complexity they would add to our parameter search. We set *E*_K_ = −105.49743 mV, calculated using the Nernst equation and the experimental configuration of [36]. *E*_Na_ was set to 50 mV, and [*Ca*^2+^] driving force was determined using the Goldman-Hodgkin-Katz (GHK) equation. Membrane resistance, *R*_m_ (inverse of ${\bar{g}}_{\text{Leak}}$, used in calculation of *I*_Leak_) and leak current reversal potential, *E*_Leak_, were free parameters in the optimization.

#### Intracellular calcium dynamics

Intracellular calcium mechanisms were approximated according to [17], with an instantaneous buffer and a single, unsaturable pump for extrusion, leading to the calcium dynamics equation:

$$\begin{array}{c} \frac{\delta {\left[Ca^{2+}\right]}_{{\text{i}}_{k}}}{\delta t}=\frac{I_{{\text{Ca}}_{k}}\times 4\times \beta }{zF\times d_{k}}-\frac{P_{\text{max}}\times 4\times \beta \times {\left[Ca^{2+}\right]}_{{\text{i}}_{k}}}{d_{k}}, \end{array}$$ (3)

where *d*_*k*_ is diameter, ${\left[Ca^{2+}\right]}_{{\text{i}}_{k}}$ is calcium concentration, and $I_{{\text{Ca}}_{k}}$ is calcium current in compartment *k*, *β* is the ratio of free to total calcium (instantaneous buffer), *P*_max_ is maximum pump rate surface density, *F* is Faraday’s constant, and *z* is the valence of calcium (2). Additional mechanisms that regulate calcium, such as slow calcium removal via the endomembrane system or mitochondria, calcium-dependent release from internal calcium stores, radial and axial diffusion (among others) undoubtedly play critical roles in regulating calcium for internal signaling within DAs. Here we used a simple and general model of the rise and decay of calcium concentration through activation of calcium channels in response to changes in membrane potential, which is sufficient for automatically generating models of the STO. We leave modeling of these other vital calcium mechanisms and their additional interactions and effects (and necessarily greater number of free parameters) for future work.

#### Ion channels

Most channel models used the Boltzmann formulation for voltage-dependent gating of activation and/or inactivation:

$$\begin{array}{c} x_{\text{inf}}\left(V\right)=1/\left(1+\left(exp\left(-\left(V-V_{\text{h}}\right)/k\right)\right)\right), \end{array}$$ (4)

where *x*_inf_(*V*) represents the steady state value of the gating variable *x* at membrane potential *V*, *V*_h_ is the half activation/inactivation voltage, and *k* is the slope of the activation/inactivation curve.

Most gating variables in our models follow the update equation:

$$\begin{array}{c} \frac{dx}{dt}=-\left[x-x_{\text{inf}}\left(V\right)\right]/\tau_{x}\left(V\right), \end{array}$$ (5)

where *τ*_*x*_(*V*) is the time constant of decay of the gating variable to steady state.

For each ion channel we introduced meta-parameters *V*_half_ and *τ*_mod_ to adjust the half-activation voltage and time constants in accordance with any reported variation in these properties among SNc DAs. We therefore used modified equations for 6 and 7, which incorporate these adjustments:

$$\begin{array}{c} x_{\text{inf}}\left(V\right)=1/\left(1+\left(exp\left(-V-V_{\text{half}}\right)/k\right)\right), \end{array}$$ (6)

and

$$\begin{array}{c} \frac{dx}{dt}=-\left[x-x_{\text{inf}}\left(V\right)\right]/\left[\tau_{\text{mod}}\times \tau_{x}\left(V\right)\right]. \end{array}$$ (7)

Time constants were also subject to a temperature-dependent scaling using a Q10 rule for those channels listed in S1 Methods having *Q*10 and *temp* parameters.

### Ion channel tuning procedures

Tuning procedures for the equations underlying gating of each ion channel model are described in detail in S1 Methods and were conducted prior to algorithmic model population optimization. This pretuning was intended to discover the range of channel parameters necessary to represent the range of single channel patch clamp recordings measured from SNC DAs. As a basis for each channel model we used existing channel models that have been incorporated into previously published single cell models of SNc DAs. In general, parameter tuning was performed for the ion channel models that had been derived from neuron types other than SNc DAs, and aimed to ensure that activity of the channels resembled recordings from SNc DAs. Section S1 Methods also provides a detailed summary of the parameter values of the model used for each channel type.

### Optimization algorithm

We used the non-dominated sorting (NS) differential evolution (DE) algorithm (NSDE). The DE implementation followed the ‘classic DE’ algorithm, or ‘DE/ran/1/bin’, with uniform jitter, *d* = 0.1, in the DE standard nomenclature [37]. We used a modified version of the BluePyOpt [38] python framework for single neuron optimization to run the algorithm.

**Fig 1:**
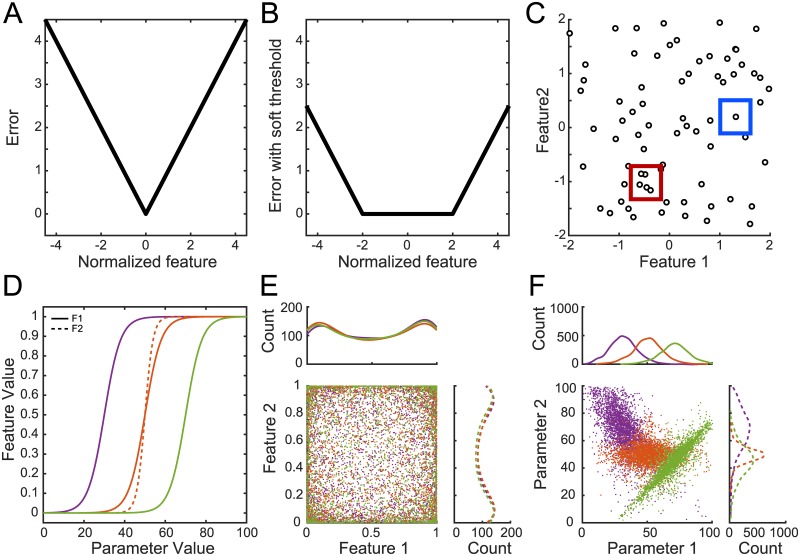
Error function description and test optimization results. (A) The traditional transfer function from normalized feature value to error value. The error value is the number of experimentally observed standard deviations from the experimentally observed mean for the model generated feature value. This method targets one ‘best’ feature value at the observed mean (function minimum). (B) Soft-thresholded error, where a number of standard deviations (in this case 2) is permitted to give equivalent error by reducing error to zero within that region. This approach considers any model within an observed range as equally plausible. (C) The decision making value when ranks are equal in the NSDE algorithm is crowdedness in feature space. Models with lower crowdedness (e.g. in the blue box) are preferred over models with higher crowdedness (e.g. in the red box). This promotes diversity of model activity and fills the full range of experimentally observed activity. (D) Sigmoidal transfer functions converting parameter values (x-axis) to feature values (y-axis) for the test optimizations. Solid lines show transfer functions for parameter 1 into feature 1; the dotted line shows transfer function for parameter 2 into feature 2 in one optimization (orange). In the other two optimizations feature 2 is calculated from a sigmoidal transfer function on either (*P*1 − *P*2) × 2 (green), or (*P*1 + *P*2)/2 (purple). (E) Test optimization result. Models shown in feature space are approximately uniformly distributed regardless of location of transfer function in parameter space. Curves at top and right in (E) and (F) are based on histograms with 100 bins. (F) Test optimization result. All model parameter sets plotted in the 2D parameter space. Distributions are Gaussian, with mean and standard deviation dependent on center and slope of the sigmoidal transfer function shown in (D).

Parameter optimizations for neuron models usually hold single mean measures or voltage traces as ideal targets, and allow for ‘acceptable’ models within 2–3 standard deviations. Here we introduced a ‘soft thresholding’ of the error function coupled with a neighborhood penalty to prevent systematic bias due to targeting exact feature values (Fig 1A and 1B). To calculate an error for each model feature, we subtracted the mean and divided by the standard deviation of experimental measures. Next, we subtracted a soft threshold from the normalized error equal to 2 standard deviations, setting negative values to 0, such that feature values within 2 standard deviations of the mean had error values of 0. We used this as the error of the non-dominated sorting differential evolution (NSDE) framework [37]. Finally, we imposed a penalty based on a measure of ‘crowdedness’ in feature space of previously selected 0 error models (Fig 1C), thus biasing the algorithm to evenly cover feature space in the 0 error region.

We verified the optimization could fully sample a target feature space, and studied how mappings from parameters to features influence the distribution of models selected by the algorithm. To do this, we simulated a simple system comprising two sigmoidal transfer functions that transformed two parameters in the range 0–100 to features in the range 0–1 (Fig 1D) and optimized using a 100 member population for 100 generations. The target feature ranges were each 0–1, so selection was driven entirely by crowdedness penalty. We performed one optimization with independent parameter to feature mappings and sigmoid functions centered at P1 = 50 and P2 = 50 (orange in Fig 1D). We performed a second optimization with the first feature calculated from a sigmoid centered at P1 = 70 and with the second feature calculated from a sigmoid parameterized by a function of P1 and P2: (*P*1 − *P*2) × 2 (green in Fig 1D). Finally, we perfomed a third optimization with the first feature calculated from a sigmoid centered at P1 = 30 and with the second feature calculated from a sigmoid parameterized by another function of P1 and P2: (*P*1 + *P*2)/2 (purple in Fig 1D). The optimization found a near uniform distribution of both feature values simultaneously (Fig 1E) despite the narrow intervals and interactions in parameter space that map to feature space. Such a mapping from parameter space to feature space intervals is one definition of ‘parameter sensitivity,’ and parameter values chosen by this test optimization form a Gaussian distribution (Fig 1F) whose mean falls at the center of the transfer functions (i.e., the most sensitive regions of parameter space). Thus this algorithm naturally samples parameter space proportional to parameter sensitivity, and provides a direct estimate of multidimensional sensitivity, including parameter interactions, using the distribution of models selected (for a discussion of why mapping a Gaussian parameter distribution into a uniform feature distribution requires a sigmoidal transfer function, and optimal information encoding consequences, see [39]). We have used this approach previously to successfully generate populations of models reflecting the range of *in vitro* excitability present in recordings from striatal medium spiny neurons [40].

Five trial runs of the evolutionary algorithm, starting from different parameter values and using different random number seeds were performed for the subthreshold optimization to ameliorate problems of local minima in the parameter search. One trial was performed of the suprathreshold optimization due to the additional computational resources required for this run. Trial-and-error was used to assess optimization metaparameters such as population size and number of generations. A smaller population and/or fewer generations than those reported were found to inadequately sample the desired feature space. Correlations were calculated with Pearson’s linear correlation coefficient, using the Matlab function ‘corr’.

### Model evaluation

Neuron model error was calculated by extracting features from the voltage response in various protocols and comparing those feature values with target values. For the subthreshold optimization a total of 7 feature values were extracted: oscillation amplitude, oscillation amplitude variance, oscillation frequency, oscillation frequency variance, input resistance and sag amplitude during a small hyperpolarization, and sag amplitude during a large hyperpolarization. Oscillation amplitude was calculated as mean peak to trough voltage difference, and frequency was calculated using mean peak to peak time interval. These were captured during a 5 s recording with no injected current, and under simulated TTX and TEA application by removing the NaT, Kv2 and BK currents. Due to the high-dimensional parameter space of the non-linear model being explored, an oscillation with mean amplitude or frequency in the target range could potentially be generated by a membrane potential fluctuating chaotically due to a combination of outlier ion channel parameters. To penalize these models with mean feature values in the correct range but with highly variable voltage fluctuations, we included variance of both amplitude and frequency in the error calculation, thereby biasing the optimization to find those models with stable (low variance) ongoing oscillations. Input resistance was calculated from response to a −10 pA hyperpolarizing current of 500 ms duration. We calculated sag amplitude in both this small hyperpolarizing current protocol, and during a 1s injection of −200 pA, by subtracting the voltage at the trough of the response from the steady state voltage during the final 5% of current injection (see S5 Fig).

For spontaneous firing optimization, we added features recorded during spontaneous pacemaking activity, with no current injection, and with the AP generating currents enabled (NaT, Kv2, and BK). From AP times during 5 s recording, we measured firing rate (FR) as the mean of the inverse of interspike intervals (ISIs) and coefficient of variation (CV) of the ISIs. From the voltage waveform of each AP, we calculated AP threshold (upward crossing of 5 mV ms^−1^ in *dV*/*dt*), AP amplitude (voltage difference between AP peak and AP threshold), AP width (duration with V above half amplitude from AP threshold), afterhyperpolarization potential (AHP) depth (V difference between AP threshold and first V minimum after the AP peak), AHP time (time from AP peak to AHP minimum), AP rise rate (mean mV ms^−1^ between crossing of AP threshold +10% of AP amplitude and AP peak −10% of AP amplitude on the rising side of the AP), and AP fall rate (mean mV ms^−1^ between crossing of AP peak −10% of AP amplitude and AP threshold + 10% of AP amplitude on the falling side of the AP). These features are illustrated schematically in S5 Fig. From the spontaneous firing recording we also calculated time differences between AP threshold crossing in the AIS relative to the soma, and in the soma relative to a non-axon-bearing proximal dendrite, to ensure AP initiation in the AIS [34, 41, 42]. Finally, we measured first AP time after the large hyperpolarization protocol, to assess rebound delay.

Error scores were calculated for each parameter set according to:

$$\begin{array}{c} \begin{array}{c} {Err}_{x}=\{\begin{array}{cc} T_{x}, & \text{if}\;T_{x}\geq 0\\ 0, & \text{otherwise} \end{array} \end{array} \end{array}$$ (8)

where *T*_*x*_ = ((|Model_*x*_ − TargetMean_*x*_|)/TargetSD_*x*_) − SoftThreshold, Model_*x*_ is the calculated value for feature *x* in the model, TargetMean_*x*_ and TargetSD_*x*_ are the target mean and standard deviation for feature *x* based on empirical observation, SoftThreshold is an offset indicating the range of feature values considered ‘acceptable’, and *Err*_*x*_ is the error score for feature *x* used by NSDE to determine model fitness. Total error was used as the decision maker between models with equal dominance rank, and a crowdedness function in feature space was used for selection when there was a full population of 0 error models and further 0 errors models were found in the offspring. Among the pool of 0 error models combined from parents and offspring, the crowdedness function sorted models by finding those with minimum Euclidean distance to nearest neighbor in feature space, selecting one of those models to be removed from the pool and placed as the ‘next most crowded model’ in a sorted list, recalculating distance to nearest neighbours for the remaining models in the pool, and so on until the pool is sorted. STO amplitude and frequency variance, sag amplitude during the small hyperpolarization, relative AP times between AIS, soma, and proximal dendrite, and AHP time from peak were not included in the crowdedness calculation, as these features were intended only to penalize models breaking these conditions.

The target means and standard deviations used for all features are shown in Table 1.

**Table 1:** Target feature mean and standard deviation values.

Feature	Target Mean	Target S.D.
All optimizations: included in crowdedness calculation		
Oscillation amplitude	17.4 mV	2.0 mV
Oscillation frequency	3.6 Hz	1.1 Hz
All optimizations: omitted from crowdedness calculation		
Oscillation amplitude Variance	0.0 mV	0.5 mV
Oscillation Frequency variance	0.0 Hz	0.25 Hz
Small hyperpolarization sag	0.0 mV	0.5 mV
Input resistance	472.0 MΩ	88.0 MΩ
Large hyperpolarization sag	34.1 mV	4.5 mV
Spiking optimizations: included in crowdedness calculation		
Firing rate	4.25 Hz	1.875 Hz
AP threshold	-42.8 mV	3.0 mV
AP amplitude	62.4 mV	5.3 mV
AP width	1.49 ms	0.29 ms
AP rise rate	63.0 mV ms^−1^	14.3 mV ms^−1^
AP fall rate	-43.2 mV ms^−1^	8.7 mV ms^−1^
AHP depth	28.6 mV	6.2 mV
Rebound AP delay	737.0 ms	375.0 ms
Spiking optimizations: omitted from crowdedness calculation		
CV ISI	0.0	0.1
AHP time	55.0 ms	22.5 ms
AP time from AIS to soma	0.5 ms	0.25 ms
AP time from soma to prox. dend.	0.5 ms	0.25 ms

#### DA neuron simulation

The optimization of subthreshold activity used 22 free parameters listed in Table 2. Each parameter set was simulated with 3 protocols: 5 s of spontaneous activity, a 500 ms small hyperpolarizing current injection (−10 pA), and a 1s large hyperpolarizing current injection (−200 pA). Each protocol was initialized at −65 mV and simulated for 5 seconds before recording to allow the model to equilibrate. The optimization of spiking activity added 14 free parameters from the 3 extra currents included to model release from simulated TTX and TEA application (NaT, Kv2 and BK), listed in Table 3. A total of 16 parameters are listed in Table 3 and were used in the spiking optimization, the additional 2 representing a dimensionality reduction of the subthreshold parameter space (see Results) into two ‘metaparameters’ (unique linear combinations of the original subthreshold parameters). Spiking activity was simulated with the 3 above protocols as well as an extra spontaneous activity protocol in which simulated TTX and TEA were applied to ensure maintenance of subthreshold oscillation features. Individual model simulations were run using the NEURON simulator (version 7.6) [43]. The ion channel model (.mod) files, parameter and feature values for the final model population, and python code for the optimization are available at ModelDB, accession number 258643 (https://modeldb.yale.edu/258643).

**Table 2:** Upper and lower bounds of parameters used in the 22-parameter subthreshold optimizations.

Parameter	Unit	Lower bound	Upper bound
${\bar{g}}_{\text{Leak}}$	S cm^−2^	1*e*^−8^	1*e*^−3^
*e*_Leak_	mV	−60	−50
${\bar{g}}_{\text{HCN}}$	S cm^−2^	1*e*^−8^	1*e*^−3^
*V*_h,HCN_	mV	−100	−70
*e*_HCN_	mV	−50	−37
${\bar{g}}_{\text{KA}}$	S cm^−2^	1*e*^−8^	1*e*^−3^
*V*_h,KA_	mV	−50	−30
*τ*_mod,KA_	a.u.	0.5	1.5
${\bar{g}}_{\text{KERG}}$	S cm^−2^	1*e*^−8^	1*e*^−2^
*V*_h,KERG_	mV	−10	10
*τ*_mod,KERG_	a.u.	0.5	1.5
${\bar{g}}_{\text{CaT}}$	S cm^−2^	1*e*^−8^	1*e*^−3^
*V*_h,CaT_	mV	−65	−45
*τ*_mod,CaT_	a.u.	0.6	1.4
${\bar{g}}_{\text{CaL}}$	S cm^−2^	1*e*^−8^	1*e*^−3^
*V*_h,CaL_	mV	−45	−25
*τ*_mod,CaL_	a.u.	0.5	1.5
*kf*_CaL_	mmol	0.0001	0.001
*P*_max_	μm ms^−1^	0	2
*β*	a.u.	0.001	0.1
${\bar{g}}_{\text{SK}}$	S cm^−2^	1*e*^−8^	1*e*^−3^
*km*_SK_	mmol	0.0001	0.001

**Table 3:** Upper and lower bounds of parameters used in the 16-parameter spiking optimizations.

Parameter	Unit	Lower bound	Upper bound
${\bar{g}}_{\text{NaT}}$	S cm^−2^	1*e*^−7^	1.0
*g*_ax,NaT_	a.u.	10.0	1000.0
*V*_h,NaT_	mV	−40	−20
*V*_h_ *h*_shift,NaT_	mV	−10	10
*V*_h_ *hs*_shift,NaT_	mV	−10	10
*V*_h_ *ax*_NaT_	mV	−10	−2
*τ*_mod,NaT_	a.u.	0.5	1.5
${\bar{g}}_{\text{Kv2}}$	S cm^−2^	1*e*^−7^	1.0
*g*_ax,Kv2_	a.u.	0.0	1.0
*V*_h,Kv2_	mV	−40	−20
*τ*_mod,Kv2_	a.u.	0.5	1.5
${\bar{g}}_{\text{BK}}$	S cm^−2^	1*e*^−8^	1.0
*V*_h,BK_	mV	−25	−5
*τ*_mod,BK_	a.u.	0.5	1.5
FPC	a.u.	−2.0	2.0
APC	a.u.	−2.0	2.0

## Results

### Novel evolutionary optimization found populations of models distributed across the full range of empirically observed features

Our aim was to produce populations of neuron models that represented the range of electrophysiological properties seen in the real DA population, and investigate relationships between ion channels involved in the characteristic subthreshold activity patterns and pacemaker firing found in these cells. Ensembles of electrophysiological models have been used for this purpose in prior approaches that vary parameters around a known seed [12, 13]. In previous work where an established parameter set for a particular model is not known, evolutionary algorithms have been shown to be highly effective at searching the high-dimensional space of ion channel parameters [44–46]. Notable studies have produced populations of parameter sets with single electrophysiological responses used as the search targets, with each parameter set representing either an individual neuron [47–50] or an average response [51–55]. Extending the framework of fitting to recordings from individual neurons, multiple optimization trials can be performed to match parameter sets to many neurons of a particular cell type, producing multiple ensembles each representing multiple fits to a different empirical recording from a specific neuron [56]. Here we use an unbiased evolutionary search of high-dimensional parameter space to find parameter sets for a new, previously unparameterized model representing a specific cell type, SNc DAs. However, during this search we incorporate a novel soft-thresholding of the error function in conjunction with a crowdedness penalty function to an otherwise standard evolutionary algorithm, differential evolution. This approach automatically accessed and sampled parameter sets from across the range of empirically observed DA electrophysiological features. Our method therefore instantiates a single optimization procedure that can find parameter sets representing models of a particular neuronal class, and then produce a large population of models, each member of which representing an instance of the class based on its empirical features, and the entire population spread across the range of all possible features. Further details are presented in the **Optimization algorithm** section of **Materials and methods**.

**Fig 2:**
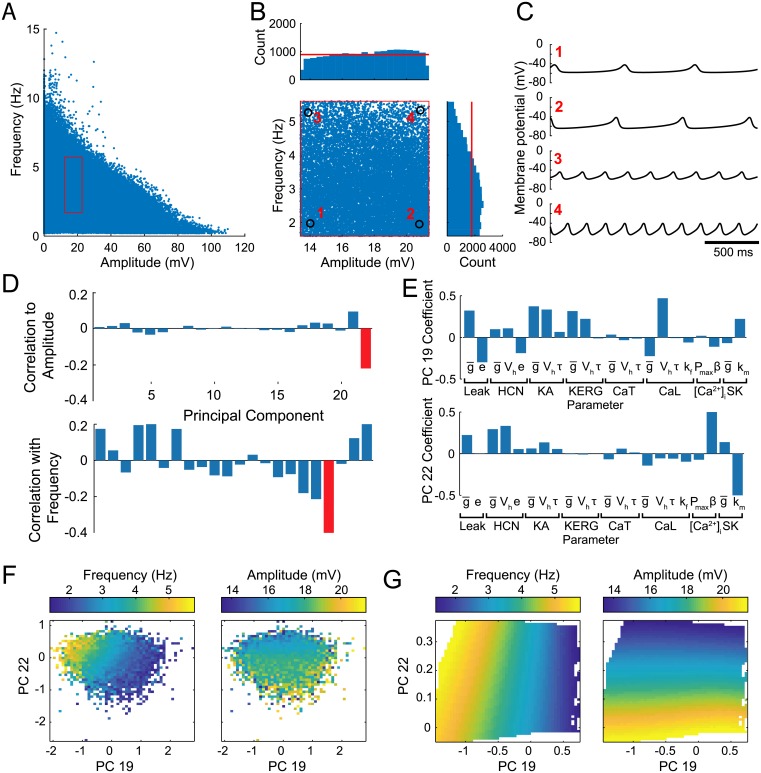
Population-based evolutionary search found models covering the range of target features. (A) STO frequency and amplitude for every parameter set simulated during the optimization. Red rectangle indicates target feature values. (B) View of red rectangle in (A) representing ‘good’ models. Histograms show distribution of values for each feature. Red lines represent uniform distribution at mean count. (C) Examples of model membrane potential during STOs with different feature values. (D) Correlation coefficients for STO features with PC scores. Red bars indicate highest correlated PC. (E) Parameter coefficients of PCs most highly correlated with STO features. (F) STO amplitude (left) and frequency (right) for models, shown in space of score for PC 19 (x-axis) and PC 22 (y-axis). Each square shows mean feature value for all models found within that segment of parameter space. Blank space indicates no models were found for that parameter combination. Models were found using 22 independently varying parameters. (G) As in (F), but showing models found during the 2-dimensional optimization using PC 19 and PC 22 as metaparameters controlling the 22 parameters according to the coefficients shown in (E).

We first used the algorithm to construct an unbiased population of multicompartment SNc DA models (described in detail in Materials and methods) with activity matching only the subthreshold characteristics of these cells, observed in the presence of TTX and TEA. TTX application is sufficient to induce an STO, and TEA application is reported to increase STO amplitude. To recreate this, we omitted currents blocked by TTX (NaT) and TEA (Kv2 and BK), and used high-voltage STO as the target [17, 57], with amplitudes between 13.4 and 21.4 mV, and frequencies between 1.6 and 5.6 Hz, according to the ranges in [57] (although STO frequency range was not explicitly specified in this study, we made an approximation from pacemaking frequencies described there). Further constraints on subthreshold activity were input resistance and sag response (see Materials and methods for full details of optimization targets). We ran our optimization algorithm on 165 CPU cores on the IBM Cloud using a population size of 165 for 5000 generations and generated 825,000 unique models in 50 hours. A wide range of STO features were generated, with amplitudes from 0–132.3 mV and frequencies from 0–18.0 Hz (Fig 2A). Within the target feature range, 18,783 models were found, distributed approximately evenly (although with fewer high frequency oscillation models found) across the full target range (Fig 2B), such that different parameter sets producing every different combination of the observed subthreshold characteristics were found (Fig 2C). To test the reliability of our optimization approach using these relatively unconstrained target features, we performed 4 additional optimization runs with different random seeds, and refer also to those results in the following analyses.

### A two-dimensional parameter plane can control subthreshold oscillation features

To describe the region of parameter space in which valid models of the adult WT DA subthreshold population were successfully found, we performed PCA on all good parameter sets from our initial optimization run, shown in S2 Fig. The distribution of each PC was well fit by a Gaussian (e.g., S2B Fig), indicating that the population of models approximates a multidimensional Gaussian in parameter space, despite STO features being more uniform in feature space (Fig 2B). Densely sampled regions were those over which features changed rapidly when metaparameters changed slowly (i.e. were more sensitive). A Gaussian distribution in metaparameter space suggests a sigmoidal transfer function from model metaparameters to model features (Fig 1).

We next investigated the sensitivity of STO features to multi-dimensional parameter changes. Two PCs, 19 and 22, accounting for relatively low amounts of variance among parameter sets (see S2A Fig), were most highly correlated with STO features (Fig 2D). Their unique coefficients (Fig 2E) represent specific combinations of parameter changes that are correlated with variation in STO features (Fig 2F). We next asked whether constraining parameters to covary along PCs 19 and 22 would reproduce the range of STO features, demonstrating the sufficiency of linear parameter transformations to control STO features throughout the target range. We repeated the optimization while restricting the 22 free parameters to vary only according to the ratios of coefficients plotted in Fig 2E, enforcing covariation of all parameters with a single metaparameter. This optimization found STO amplitudes from 0–26.4 mV and STO frequencies from 0–14.6 Hz. The target region of feature space contained 38,270 models, and when STO features were mapped into this plane of control (Fig 2G), their variation fell along orthogonal axes. This analysis shows how control of electrophysiological features can be achieved within low dimensional projections of a high dimensional parameter space.

**Fig 3:**
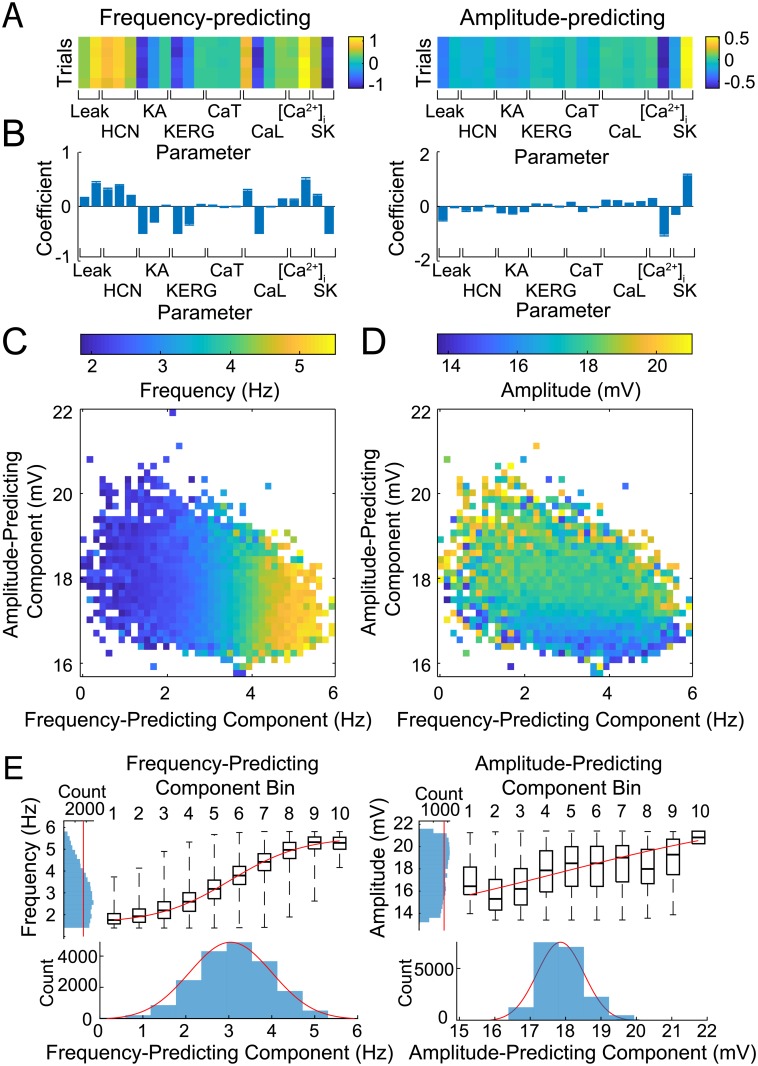
PLSR identified predictors of features. (A) Parameter coefficients from PLSR against STO frequency and amplitude. Rows indicate PLSR performed on output parameter sets from 5 independent trials of the optimization algorithm using different random seeds. (B) Mean coefficient from the 5 trials for each parameter. Error bars (not visible due to small size) show standard deviation. (C) STO frequency for models shown in the space of predicted amplitude and frequency for that parameter set, according to the PLSR coefficients. Color shows mean STO frequency for all models within a particular bin of predicted frequency and amplitude space. Empty squares indicate no models in that range of predicted feature space. (D) As in (C), but with color showing model STO amplitude. (E) All models sorted by predicted feature values according to PLSR coefficients, and binned into 10 equal-width bins of predicted STO feature values. Boxplots show actual feature values of all models in that bin. Centre line shows median, box shows interquartile range, and whiskers show range. Red lines across boxplots show best fitting sigmoid functions calculated using least squares error. Outside histograms show distribution of predicted STO feature values (horizontal) and actual STO feature values (vertical). Red lines in horizontal histograms show Gaussian fits of models in parameter space, as in Fig 2E. Red lines in vertical histograms show uniform distribution at mean count.

Having shown that two PCs can be used to control STO over a broad range of features by constraining ion channel parameters to vary in specific ratios, we next aimed to find optimal controllers of these features. We employed partial least squares regression (PLSR) to define these controllers. PLSR is a multivariate regression method, which finds coefficients (ratios) of independent variables that optimally predict dependent variables, and has been used previously to understand the relationship between ionic conductances and action potential features in cardiac models [12, 58–60]. We applied PLSR to the parameter sets identified by our algorithm and determined the linear transformations (controllers) of parameter sets that were most reliable at predicting changes in STO features. We performed PLSR independently on the 5 model populations generated from the 5 optimization runs. PLSR found controllers (linear operators) that most consistently mapped parameter sets to either STO amplitude (Fig 3A, left) or frequency (Fig 3A, right). Fig 3A shows optimization trials sorted by similarity of PLSR coefficients, revealing consistent controllers of function across trials.

Mean coefficients for the amplitude-predicting component (APC) had large magnitudes for ${\bar{g}}_{\text{Leak}}$, *β*_Cai_, and *k*_m,SK_. These coefficients are similar to those found for PC22 in Fig 2E, but without the large coefficients for ${\bar{g}}_{\text{HCN}}$ and *V*_h,HCN_, indicating that some parameter coefficients found in the PC do not enhance reliability of prediction of the resulting model activity (of course, reliability of prediction was not a constraint of PCA). The mean frequency-predicting component (FPC) had large coefficients for ${\bar{g}}_{\text{KA}}$ and *V*_h,KA_, ${\bar{g}}_{\text{KERG}}$ and *V*_h,KERG_, ${\bar{g}}_{\text{CaL}}$ and *V*_h,CaL_, *β*_Cai_, and *k*_m,SK_. Remapping STO feature values from our initial optimization into the control plane defined by the APC and FPC revealed reliable prediction of STO frequency (Fig 3C and 3E left), but less reliable prediction of STO amplitude (Fig 3D and 3E right). This finding was reinforced by an R^2^ statistic value of only 0.063 for the APC, but a larger R^2^ statistic value of 0.653 for the FPC (S2A and S2B Fig).

To investigate the reliability of these axes of control further, we divided models into 10 bins from across the range of predicted values for each STO feature, and the actual feature values of models from each bin are shown in the boxplots of Fig 3E. The progression of median values for actual features within each bin very closely follows a sigmoid for STO frequency, as shown by the sigmoid fit to the bin medians (Fig 3E left). For STO amplitude, this fit stretched the sigmoid to a near linear controller, and the larger variances in boxplots for STO amplitude (Fig 3E right) indicate weaker control of this feature using the PLSR coefficients than for STO frequency. The vertical histograms in Fig 3E show the distributions of predicted feature values for STO frequency and amplitude, and the horizontal histograms show a Gaussian distributions of models within parameter space (as in S2E Fig along PCs). A Gaussian distribution in parameter space and a near uniform distribution of STO frequency reinforces that the model transfer function within the control plane was sigmoidal, suggesting that we indeed have uncovered an axis close to the optimal controller of STO frequency. The parameter space around the steepest slope of this sigmoid identifies a region of highest sensitivity, and our optimization technique therefore performed an implicit sensitivity analysis by centering our model population around this region, while identifying viable parameter sets that fill the target feature range.

The low prediction precision of STO amplitude highlights a limitation of our linear model of parameter changes required to modulate amplitude. Because our model consists of many non-linear components, non-linear metaparameters could act as controllers and potentially facilitate greater prediction accuracy (indeed, performing second-order polynomial regressions of parameters against STO features obtained increased R^2^ statistic measures of 0.907 for the FPC and 0.397 for the APC (see S2C and S2D Fig)). Our linear controllers, while not fully able to predict the features, were nevertheless strikingly sufficient for recovery of the full range of activity of that feature. Our intention in this first stage of optimization was to identify a lower dimensional region of parameter space that could linearly produce accurate subthreshold activity, suggesting that parameters for subthreshold ion channels could be meaningfully constrained by controlling metaparameters. We next aimed to use this dimensionally-reduced control plane as a basis for generating a population of spiking SNc DA models. We therefore used the controlling metaparameters of STO features derived from linear regression as a plane from which to extend the parameter search for spiking activity.

### Spontaneous firing features occupying the target range were discovered starting from the linear plane of STO control

The precise relationship between STO and the spontaneous, pacemaker firing observed in SNc DAs is yet to be fully characterized. It has been hypothesized that the STO might underlie the depolarization leading to pacemaker firing, with the STO driving the voltage until the spiking currents activate [17], but this idea has been challenged by observations that the calcium current, which forms the basis of the STO depolarization, does not necessarily dictate pacemaker frequency. Instead, this frequency may be more reliant on subthreshold sodium channel activation [19]. Multiple sources of depolarization must interact during the spontaneous interspike interval, resulting in a complex and potentially compensatory framework dictating pacemaker frequency [18, 61]. The regularity of pacemaker firing also varies in SNc DAs with synaptic inputs [41], current injection [41, 62], ion channel blockade [63, 64], and juvenile development [36], and each has been demonstrated to modulate regularity of firing. Again, multiple interacting currents may lead to particular regimes that make a neuron more or less susceptible to irregular or burst spiking despite normal pacemaker function. A major aim of this work was to facilitate precise predictions concerning specific ratios of ion channel properties that can regulate features within each of the SNc DA activity modes. As such, we next aimed to utilize our novel optimization framework to generate a population of models within the full empirical range of SNc DA spontaneous electrophysiological characteristics. This would allow investigation of the relationship between subthreshold and suprathreshold regimes, and their currents.

We performed an optimization with 3 channels added to the model, NaT, Kv2 and BK, each of which was absent in the previous subthreshold condition (simulating blockade by TTX and TEA). Channel parameters were $\bar{g}$, *V*_half_ and *τ*_mod_, and control parameters were added for scaling the $\bar{g}$ of NaT and Kv2 in the AIS, shifting *V*_half_ for NaT in the AIS, as well as for shifting the *V*_half_ of fast and slow inactivation relative to activation for NaT, in order to allow the optimization to adjust the NaT window current (see Materials and methods and S1 Methods). In our analysis of the subthreshold response of the model, we uncovered a 2D linear plane capable of reproducing the full range of STO frequencies and amplitudes. Here we used this dimensionality reduction of 22 parameters to 2 metaparameters as a basis for the addition of the AP generating currents, substantially reducing the complexity of the parameter space to be explored for spiking models. This approach enabled the AP firing models to have STO features from potentially any point in the frequency and amplitude ranges, and ensured that they had reasonable input resistance and sag amplitudes, as these features were also constrained in the subthreshold search. We simulated a spontaneous firing protocol, and small and large hyperpolarization protocols with all currents, as well as a spontaneous subthreshold activity protocol maintaining simulated TTX and TEA, to guarantee that appropriate subthreshold response would not be lost by the addition of spiking currents. While many measurements of rodent SNc DA AP features have been made in prior publications, [36] reported AP features such as threshold, amplitude, width, rise slope, decay slope, AHP depth and rebound delay from a consistent set of experimental protocols, so we chose to use this single source for target feature values for the spontaneous firing protocol. Although mean ISIs were reported in [36] as 0.92±0.54 s, we chose to allow AP firing rates between 0.5 and 8.0 Hz, approximating the range reported elsewhere [65, 66] under varying experimental conditions. Additional features included constraints on relative timing of AP onset in the AIS, soma, and proximal, non-axon-bearing dendrite, to ensure AP initiation occurred in the AIS [34, 41, 42]. This optimization had 16 free parameters and 23 target features (see Materials and methods for full details of parameter and feature ranges). We ran the optimization with a population size of 165 across 165 CPU cores on the IBM Cloud for 10,000 generations, testing 1,650,000 parameter sets in 600 hours. This search found 2,047 models matching all target features simultaneously. A second optimization, performed for comparison, used the full original ranges of all parameters from from Tables 2 and 3 (excluding of course the APC and FPC metaparameters), resulting in 36 free parameters. When similarly run for 10,000 generations, this optimization was unable to find any models matching all target features simultaneously, highlighting the utility of building spiking models outward from a subspace of subthreshold parameters. We then applied additional constraints to check the validity of discovered models using constraints approximated from observations in the SNc DA literature and experimental protocols that models were previously not tested on. Applying these constraints during the optimization would have increased simulation time excessively, so we based the optimization on features extracted from a minimal set of protocols and applied these contraints *post-hoc*. We tested the response of each model to a 100 pA current injection, and eliminated models from the final population that fired at above 30 Hz in that condition, constraining the population to a reasonable range for SNc DA neurons [41]. We further restricted the final population by removing models that produced unusual responses under simulated channel blockade experiments from which we were unable to automatically calculate feature values. This resulted in a final population of 727 models meeting all criteria. Parameter values for this final population are shown in S3 Fig.

**Fig 4:**
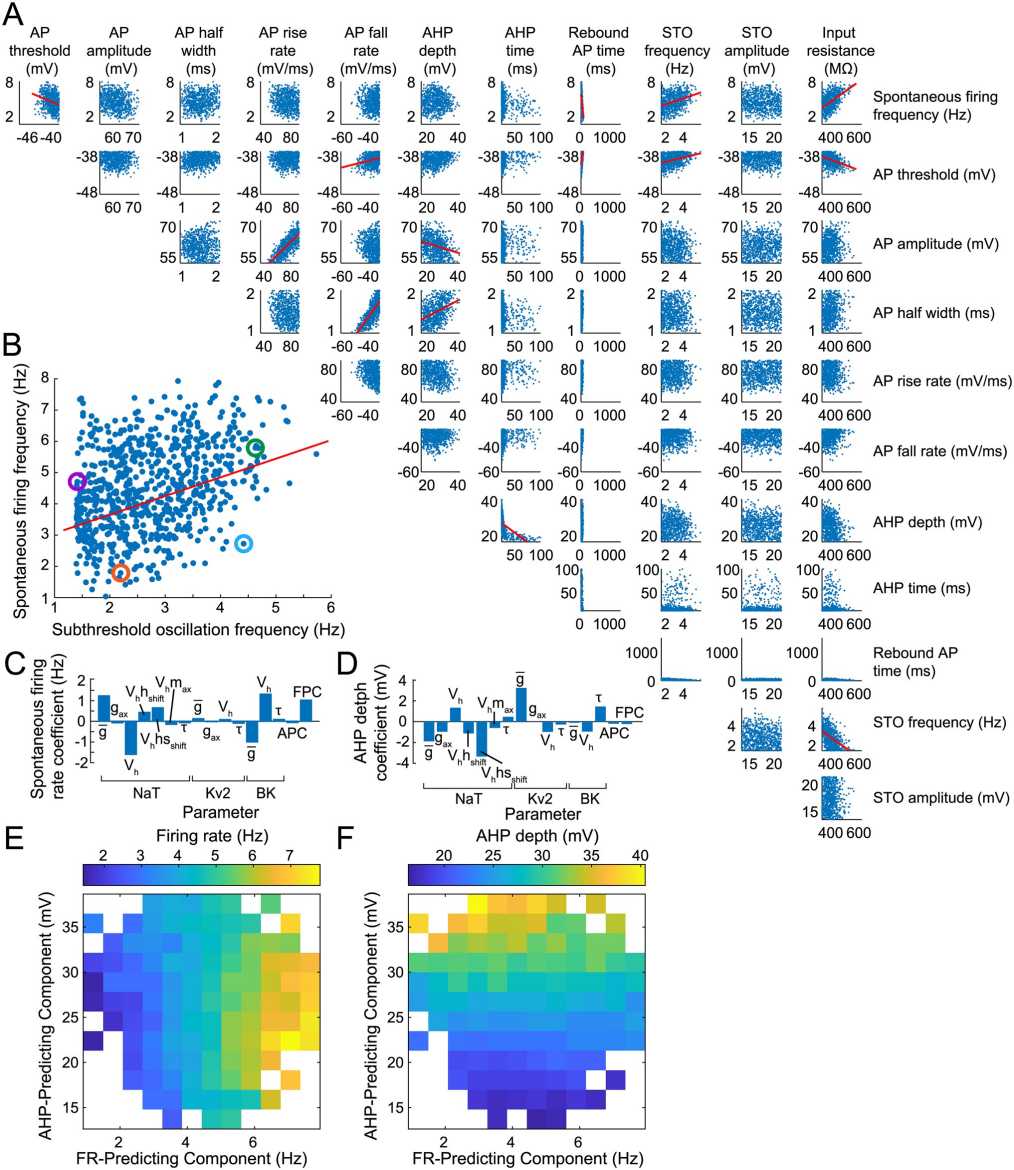
Spiking optimization features. (A) Scatter plots of feature pairs for all 727 models present in the final population. Red lines show correlations with coefficients above 0.25. (B) Zoom on STO frequency vs. spontaneous FR. Colored circles correspond to colored traces in Fig 5. (C) PLSR coefficients for spontaneous FR. (D) PLSR coefficients for AHP depth. (E) Spontaneous FR for models shown in the space of predicted spontaneous FR (x-axis) and AHP depth (y-axis) for each parameter set, according to the PLSR coefficients. Color shows mean spontaneous FR for all models within a particular bin of predicted FR and AHP depth. Empty squares indicate no models in that range of predicted feature space. (F) As in (E), but with color showing model AHP depth.

The relationship between frequencies of STO and spontaneous firing in SNc DAs is unknown. One observation [19] suggests a positive linear correlation, such that neurons with a fast STO have a fast spontaneous firing rate and *vice versa*, albeit with potential outliers. Feature values of the model population are shown in Fig 4A for 12 of the target features. Among our model population, we found a correlation of 0.34 (*p* < 0.001) between STO and spontaneous firing frequencies (Fig 4B), but we also found outliers across the full feature range. Other feature values were spread across the majority of the target ranges, with one exception: rebound spike times were constrained to a narrow, low range. Delay to first spike after a hyperpolarization is known to depend on the kinetics of the KA current [4], which were constrained to covary completely within the 2D plane of STO frequency and amplitude controllers, such that the optimization did not have the opportunity to fully explore parameters contributing to this feature. The population therefore only represents the portion of SNc DAs with a fast rebound response, and leaves open a possible mechanism to regulate this important timing property among SNc DAs [67]. Albeit, this is a common property of SNc DAs, which have generally faster rebound from hyperpolarization than DAs in the ventral tegmental area (VTA). Limiting the population to fast rebound demonstrates that the dimensionality reduction approach used here may constrain parameter sets to covary in a specific way, and potentially restrict feature value variation in subsequent optimizations of the population.

**Fig 5:**
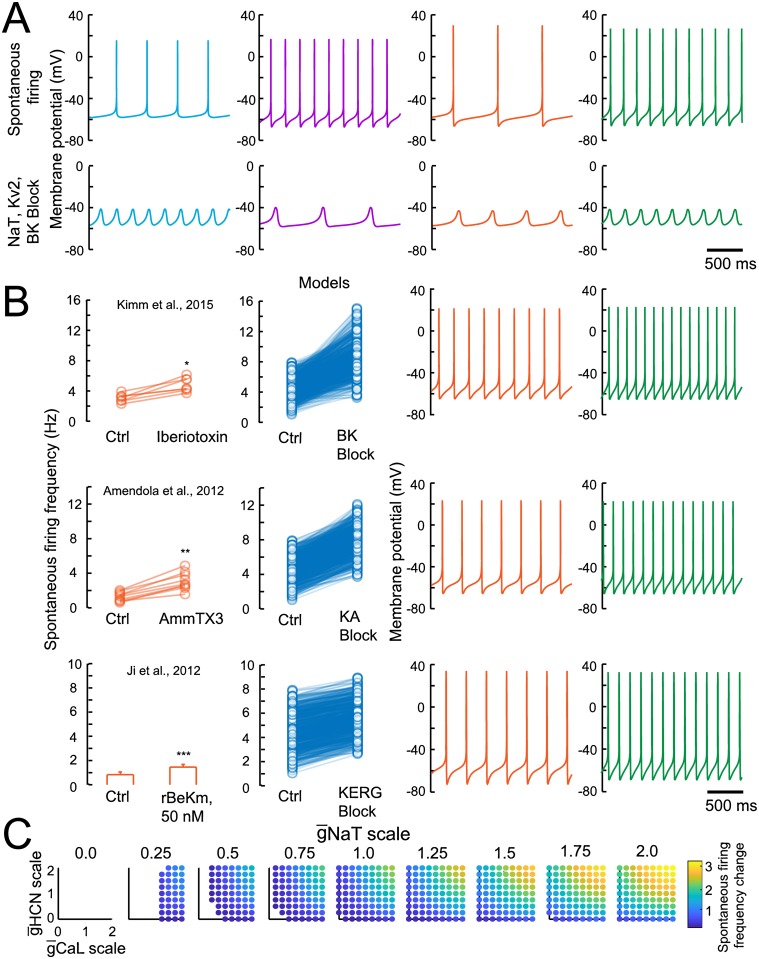
Spiking model channel perturbation results. (A) Spontaneous firing and STO voltage traces from 4 example parameter sets. Colored traces correspond to models circled in Fig 4B. (B) Effect of blocking potassium currents on spontaneous firing frequencies. Left plots with orange points are recreated from Figure 3C of [64] (BK Block), Figure 2D of [24] (KA Block), and Figure 1F of [10] (KERG Block). Right plots with blue points show effects in all good models. Points paired by lines indicate each model. Traces correspond to the orange and green models shown in (A), for each of the channel block conditions. (C) Effect of positive and negative scaling of depolarizing conductances (NaT, CaL and HCN) on spontaneous firing frequency. Each square shows a different scaling factor applied to ${\bar{g}}_{\text{NaT}}$, with 9 scaling factors ranging from 0 to 2 applied to all 727 good models. Within each square, x-axis shows scaling of ${\bar{g}}_{\text{CaL}}$ and y-axis shows scaling of ${\bar{g}}_{\text{HCN}}$. Colors indicate mean change in frequency from default value for all 727 models.

We explored how parameters related to the features of the spontaneous firing by performing PLSR on the final population (shown for all spiking features in S4 Fig). Parameter coefficients for predicting spontaneous FR are shown in Fig 4C. The largest coefficient corresponds to *V*_half,NaT_ and is negative, indicating that active sodium current at lower membrane potentials was the most reliable way to increase FR. A prominent positive coefficient for ${\bar{g}}_{\text{NaT}}$ further indicates the crucial role of sodium current in determining FR. Another prominent positive coefficient corresponded to the subthreshold oscillation’s FPC, revealing that modulating subthreshold currents along this metaparameter both increases STO frequency and reliably increases spontaneous FR. Note that because only the FPC and APC parameters affect the STO, the remaining coefficients also indicate the parameters that cause the divergence between STO frequency and spontaneous FR seen in Fig 5B. The remaining two prominent coefficients corresponded to ${\bar{g}}_{\text{BK}}$ and *V*_half,BK_, while very low coefficients were found for all Kv2 parameters, implicating BK current more strongly in dictating FR than Kv2 current. Blockade of either of these currents has been found to significantly elevate spontaneous FR in SNc DAs [64], whereas only one of these currents was found to reliably modulate spontaneous FR within our population of models.

Correlation coefficients above 0.25 were found for only a handful of additional feature pairs (indicated by red lines in Fig 4A) with the strongest correlations (above 0.7) appearing only between features already expected to covary, such as AP rise rate and AP amplitude, or AP fall rate and AP width. Most feature pairs had correlations under 0.25, indicating that the crowdedness function used in this optimization approach had encouraged spread across combinations of features, limiting correlations among feature pairs where possible. AHP depth is an example of a feature found to be uncorrelated with FR, and the PLSR coefficients predicting AHP depth are shown in Fig 4D. The population of models can be arranged in terms of their predicted FR and AHP values, which represents a view through parameter space, shown in Fig 4E and 4F. The colors in Fig 4E and 4F show mean feature values from models within a particular part of the predicted feature space (FR and AHP). The consistent variation of these features across this projection of parameter space is another example of our analysis of a population-based parameter search discovering parsimonious dimensions of control of electrophysiological features within the high-dimensional parameter space of a non-linear model.

Next, we examined the generalizability and robustness of the discovered models by performing blocking experiments on individual channels and observing the effects on spiking activity.

### Discovered models show robustness and compensation under ion channel perturbation

We simulated spontaneous activity under conditions in which each current was blocked by reducing $\bar{g}$ to 0. Each blockade experiment was recorded for 10 s, and features such as FR in the final 5 s were extracted for all 727 models in the final population. First, we observed that spontaneous firing did not emerge reliably when blocking Kv2 in these models (not shown), indicating that the optimization had found a region of parameter space in which the Kv2 current was essential for generating spontaneous firing.

Examples of spontaneous FR and STO voltage traces are shown in Fig 5A, from four models from different points of the feature space (corresponding to the colored circles in Fig 4B). Blockade of the BK, KA and KERG potassium currents (Fig 5B) produced a reliable increase in spontaneous firing rate across the model population, consistent with empirical observations [10, 24, 64]. The traces in Fig 5B show the effects of channel blockade on the orange and green example models shown in Fig 5A, indicating modulations of AP shape and the robustness of the spontaneous firing.

**Fig 6:**
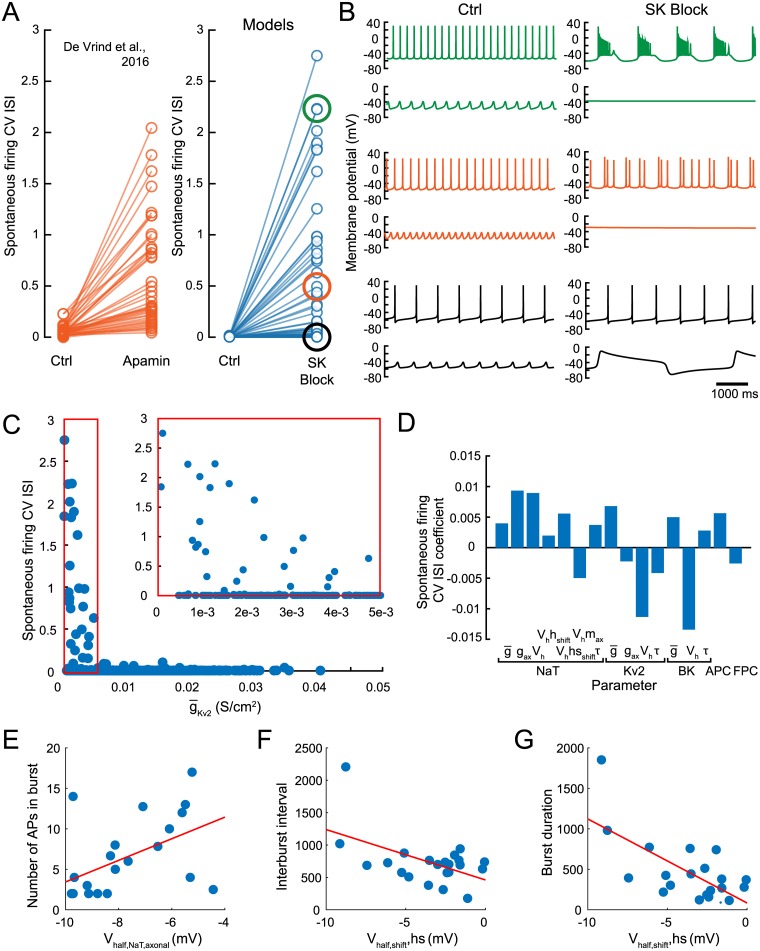
Burst susceptibility of models under SK block. (A) CV ISI during normal spontaneous firing (Ctrl) and with SK conductance reduced to 0 (SK Block). Left plot with orange points is approximated from Figure 2A of [32]. Right plot with blue points shows the population of models. Black, orange and green circles indicate CV ISI values of traces shown in (B). (B) Example voltage traces for spontaneous firing (top) and STO (bottom) in the normal (Ctrl) and SK Block conditions, for parameter sets demonstrating 3 behaviors: large increase (green), small increase (orange) and no change (black) to CV ISI under SK Block. (C) Kv2 conductance (x-axis) vs. spontaneous firing CV ISI (y-axis). Inset shows zoom in on red boxed region (low ${\bar{g}}_{\text{Kv2}}$). (D) PLSR coefficients for CV ISI for all parameter sets with ${\bar{g}}_{\text{Kv2}}<0.005$. (E-G) Scatter plots of 22 models in which all burst characteristics could be calculated, showing highest correlated parameter (x-axes) with each burst characteristic (y-axes). Each point is a parameter set.

Because blockade of NaT eliminated spiking, we aimed to more thoroughly examine the effects of perturbations to depolarizing currents. We scaled ${\bar{g}}_{\text{NaT}}$, ${\bar{g}}_{\text{CaL}}$ and ${\bar{g}}_{\text{HCN}}$ between 0 and 2 times their values for each good model, at intervals of 0.25. This led to 9 scaling factor values for each of these 3 parameters, giving 729 combinations. We simulated spontaneous activity for all 727 models in the final population, for each of the 729 scaling factor combinations, calculating 529,983 sets of features. We measured the firing rate change as a multiplier from baseline for each individual, and calculated mean change for each scaling factor combination across all individuals. Circle colors in Fig 5C shows mean firing rate change, where an empty space indicates spontaneous firing was eliminated in all 727 individuals. Across the 9 squares, ${\bar{g}}_{\text{NaT}}$ is scaled from 0 to 2, with ${\bar{g}}_{\text{CaL}}$ scaled horizontally from 0 to 2 and ${\bar{g}}_{\text{HCN}}$ scaled vertically from 0 to 2 within each square. The left square, with full blockade of NaT, shows no activity among any individual regardless of any change to ${\bar{g}}_{\text{CaL}}$ or ${\bar{g}}_{\text{HCN}}$. The bottom left corner of the center square, with default NaT values, shows that full blockade of both CaL and HCN was also sufficient to eliminate AP generation in all individuals. A common feature across squares is that change to spontaneous firing rate by perturbation of one depolarizing current can be compensated for by an opposing perturbation of other depolarizing currents, indicating degeneracy among sources of depolarization. This type of degeneracy between currents has been shown to be useful for robust activity generation in neurons [61]. For example, firing rates can be maintained during gradual increase of ${\bar{g}}_{\text{CaL}}$ (moving right within a square) by decreasing ${\bar{g}}_{\text{HCN}}$ (moving down within a square), mirroring SNc DA juvenile development, during which a compensatory reduction in HCN current accompanies a developmental increase in CaL current [68]. We found that the mean effect of blockade of CaL was a reduction in spontaneous FR (baseline: 4.3527± 1.40474 Hz; CaL block: 0.0549± 0.3389 Hz), consistent with some experimental results [16, 68, 69], but in contrast to [19], where downregulation of CaL sufficient to eliminate [*Ca*^2+^] transients during APs was found to have no mean effect on spontaneous FR. The resilience of DA models to CaL blockade has previously been shown to require specific balance between NaT and CaL parameters [18, 70], with models with low CaL to NaT conductance ratios entering the regime found among most of the final population here. While the final population of models generalized to the effects of multiple experimental perturbations, future optimizations with additional constraints may allow construction of a model population that reflects a finer balance among parameters, much as prior experimental and modeling results have indicated are likely present in the real SNc DA population. The final channel blockade experiment targeted SK current and did not produce a reliable effect on spontaneous FR (not shown). Instead, an increase in CV ISI (Fig 6A) appeared inconsistently, thus replicating empirical observations that SK modulation can, but does not necessarily, induce CV ISI increases in SNc DAs [32, 63, 71].

### Model burst susceptibility under SK block is determined by AP repolarizing potassium currents

Our models were deterministic, so modulation of firing regularity must result from fundamental alterations to mechanisms responsible for the pattern of spontaneous activity. Large increases in CV ISI manifested as prolonged bursts with multiple spikes (Fig 6B, green), while small increases in CV ISI manifested as doublet or triplet bursting activity (Fig 6B, orange). Most individuals in the final population did not show an increase in CV ISI under SK block (Fig 6B, black), despite the presence of characteristic plateau potentials emerging in some models under simulated TTX, TEA and apamin application (Fig 6B, black). Note that plateau potentials under SK block in subthreshold simulations was neither necessary nor sufficient for inducing bursting activity, leading us to next examine the conditions leading to increased bursting susceptibility under SK blockade.

A low value of ${\bar{g}}_{\text{Kv2}}$ was a necessary condition for burst generation under SK block (Fig 6C), with CV ISI only increasing under SK block in models with ${\bar{g}}_{\text{Kv2}}<0.005\;\text{S}\;{\text{cm}}^{-2}$ (termed low-Kv2 models). This condition was not sufficient for burst generation, however, with some low-Kv2 model CV ISI values remaining low (Fig 6C, inset). We performed PLSR on all low-Kv2 model parameter sets, regressing against CV ISI under SK block to determine the directions in parameter space that most reliably lead to bursting activity. The largest coefficients, shown in Fig 6D, corresponded to *V*_half,BK_ and *V*_half,Kv2_ (both negative) and indicate that higher voltage thresholds for activation of repolarizing potassium currents leads to a higher likelihood that a model is susceptible to bursting under SK block. The next most prominent parameter coefficient was for the conductance scaling parameter for NaT in the AIS, meaning that models with strong NaT in the AIS relative to the soma are more likely to burst. This was followed by *V*_half,NaT_, further highlighting the voltage threshold of spike-generating currents as a strong regulator of burst susceptibility under SK.

We next calculated feature values for burst characteristics: mean number of APs in a burst, interburst interval, and burst duration. Of the 31 models with CV ISI above 0.2 under SK block, all burst feature values could be calculated for 22 (burst features could not be calculated for 9 models with irregular spiking, resembling that of the orange trace in Fig 6B). Fig 6E–6G show these characteristics. The parameters with the highest correlation with each feature were all related to the NaT channel. Number of APs per burst was most highly correlated with *V*_h_ *ax*_NaT_ (0.501, Fig 6E), and interburst interval and burst duration were each most highly correlated with *V*_h_ *hs*_shift,NaT_ (−0.519 and −0.671, shown in Fig 6F and 6G, respectively). This indicates that sodium channel slow inactivation plays a significant role in terminating and resuming bursts, resembling the empirical finding that slow inactivation of sodium determines depolarization block during depolarization-induced bursts in SNc DAs [72, 73]. These observations represent a set of experimentally testable predictions surrounding burst susceptibility under SK block: first, that bursting only emerges in SNc DAs with a lower magnitude of Kv2 current; second, that among those cells with low Kv2 current, half activation voltage of rectifying potassium channels such as Kv2 and BK will indicate the degree of spiking irregularity generated under SK block; and third that, in this condition, burst properties are correlated with the voltage at which NaT current activates and inactivates.

### Ion channels occupy fundamentally different states during STO and spontaneous firing

**Fig 7:**
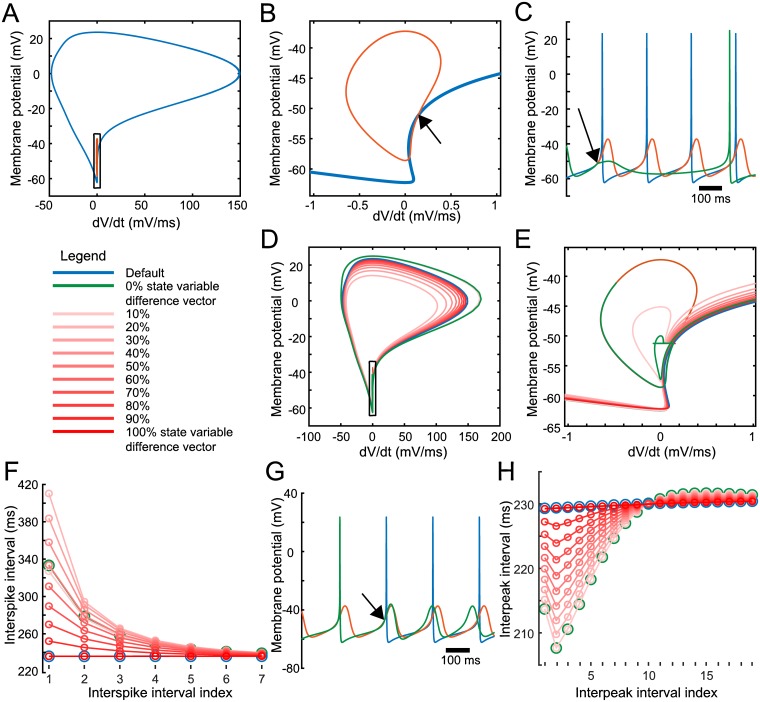
Spontaneous firing and STO interaction. (A) Phase plane of membrane potential for spontaneous firing (blue) and STO (orange) for one model with similar frequencies in both regimes. (B) Zoom in on black boxed region of (A). Arrow shows point of crossing at which membrane potential and rate of membrane potential change are identical in both regimes. (C) Voltage traces for spontaneous firing (blue), STO (orange), and STO with spiking currents (NaT, Kv2, BK) activated at point of identical voltage (green). Arrow indicates the point at which spiking currents are activated in the green trace, and the orange and green traces diverge. (D) Phase plane of membrane potential for spontaneous firing (blue), STO plus activated spiking currents (green), and STO plus activated spiking currents plus fractional addition of the state variable difference vector (shades of red). Colors correspond to the key shown to the left. (E) Zoom in on the black boxed region of (D). (F) Interspike intervals for the first 7 ISIs calculated from the first 8 spike times after spiking current activation. (G) Voltage traces for spontaneous firing (blue), STO (orange), and spontaneous firing with spiking currents (NaT, Kv2, BK) deactivated at point of identical voltage (green). Arrow indicates the point at which spiking currents are deactivated in the green trace, and the blue and green traces diverge. (H) Interpeak intervals for the first 19 interpeak intervals calculated from the first 20 oscillation peak times after spiking current deactivation. Colors correspond to the key shown in (F).

Above, we explored the relation between SNc DA spontaneous activity in the sub- and supra-threshold regimes, finding a model population with a positive correlation between oscillation and spiking frequencies (Fig 4B). Regression analysis indicated that both subthreshold and suprathreshold current parameters influence spontaneous firing rate (Fig 4C). However, the interaction between these two electrophysiological phenomena remained obscure, even with complete access to model parameters and feature measurements. To further elucidate this relationship, we investigated the voltage trajectories of both activity patterns in the models. In the phase plane of *V* and *dV*/*dt*, both subthreshold oscillation and spontaneous APs form loops (Fig 7A shows one model with similar frequencies for both regimes). A smaller amplitude and slower subthreshold oscillation (Fig 7A, orange) is naturally nested within the larger and faster AP (Fig 7A, blue). We noticed that these trajectories drew nearest each other on their rising phases while the membrane potential accelerated into the AP within the spiking regime and the subthreshold oscillation decelerated towards its peak, crossing in some cases, indicated by the arrow in Fig 7B. The two regimes therefore had identical voltage and rate of voltage change at certain phases. Given this observation that ongoing activity was most similar at these points, we first asked if inserting spiking currents through simulated instantaneous TTX and TEA washout at this precise phase in the subthreshold regime would cause the model to transition immediately into its normal spontaneous firing mode?

We performed this experiment by simulating the model shown in Fig 7A and 7B for 5 s in the subthreshold regime, with ${\bar{g}}_{\text{NaT}}$, ${\bar{g}}_{\text{Kv2}}$ and ${\bar{g}}_{\text{BK}}$ set to 0. At the detected crossing of the voltage indicated by the arrow in Fig 7B, we simulated instantaneous washout of TTX and TEA by setting the spiking conductances to the default values for that model then continued the simulation. The resulting membrane potentials, shown in green in Fig 7C, diverged from both the normal subthreshold oscillation (orange) and spontaneous firing (blue) trajectories, reaching a lower subthreshold peak than the normal oscillation and remaining hyperpolarized for over 500 ms before generating an AP. Due to the ongoing maintenance of the voltage in the subthreshold regime prior to spiking current activation, gating variables for the activation and inactivation of sodium and potassium channels were not in a configuration to immediately enter an AP, as they would have been at that phase had an ongoing spontaneous firing regime been present. Of course, all internal variables of the model are different during the subthreshold and spiking regimes. We next examined the impact of modulating this state difference on spike timing after spiking current activation.

The difference in internal state between the two regimes at the point of crossover in the phase plane was calculated by the difference between every variable in the model, from all channels across all compartments, including voltage and calcium variables, leading to a 336-dimensional ‘state variable difference vector’. Simultaneous activation of the spiking currents and subtraction of this difference vector across all variables in all compartments of the model readily shifted the model from the subthreshold regime to the spiking regime, resulting in a near perfect match of the remaining simulation to the default spiking regime voltage trajectory (compare blue and red traces in Fig 7D and 7E).

Next, we performed a sequence of similar experiments by sequentially restoring fractions of the difference vector, at 9 intervals from 0.1 to 0.9. Fig 7D shows the resulting voltage trajectories with Fig 7E showing a zoom in on the black boxed region of Fig 7D. In Fig 7E, the hyperpolarization and delayed AP found previously with no state variable adjustment (green in Fig 7C) can be seen in the green trace as the additional loop prior to AP onset (AP onset occurs when the trajectory heads upwards and to the right into the AP). This loop, and subsequent delayed AP, also occurred when subtracting 10% of the difference vector (lightest red trace), but at 20% a transition occurred, and the trajectory immediately proceeded into the AP with no prolonged hyperpolarization. Subsequent subtraction of increasingly large fractions of the difference vector (lighter to darker red traces in Fig 7D and 7E) moved the resulting trajectory towards the normal spiking trajectory (blue trace in Fig 7D and 7E).

We found increased ISIs immediately after spiking current onset (Fig 7F), which gradually decreased over the first 6 APs, increasing the firing rate towards the normal frequency. However, with either 0% or 10% of the difference vector subtracted, the ISIs were actually closer to the normal values than with 20% of the difference vector subtracted, which resulted in the strongest frequency modulation and increased the first ISI to nearly double its normal value. Further increasing the fraction of state variable difference vector subtracted gradually decreased ISIs towards normal values. ISIs decay to within 5% of the original ISI baseline after 5 ISIs, a process taking around 2 s in the default case (green in Fig 7F), which also includes approximately 500 ms before AP onset.

The instantaneous transition from spontaneous firing to subthreshold oscillation through simulated TTX and TEA application can also be performed *in silico*. Here, we simulated the model for 5 s with spiking currents, then set spiking conductances to 0, at the time indicated by the arrow in Fig 7G. The green trace in Fig 7G shows that the model immediately enters the subthreshold oscillation regime, but with a frequency modulation relative to the normal ongoing subthreshold oscillation, shown in orange. Again, we subtracted incremental proportions of the state variable difference vector, this time reflecting the transition from the spontaneous firing regime to the subthreshold oscillation regime, and plotted the interpeak intervals (IPIs) after spiking current block (Fig 7H). In the case without difference vector subtraction, we observed a smaller first IPI, which became still smaller for the second IPI, then began relaxation towards the normal frequency, indicating a transitional period in which the neuron adapted to the new regime. IPIs remained 5% below baseline for 5 IPIs, taking approximately 860 ms to increase to baseline. Incrementally subtracting increasing fractions of the difference vector led to incremental increases in IPIs, until the activity was indistinguishable from the normal STO with 100% of the state variable difference vector subtracted, instantaneously transitioning the neuron from the spiking regime to the subthreshold oscillation regime.

These results indicate that the alternate configuration of state variables attained during subthreshold oscillation compared with spontaneous firing leads to a temporal lag of spiking activity caused by a prolonged hyperpolarization upon activation of spiking currents for this parameter set. This hyperpolarization did not occur if state variables were adjusted by subtracting 20% of the difference vector, and doing so instead led to a modulated (slower) frequency of spontaneous firing, despite this parameter set having ongoing oscillations at a similar frequency to the spontaneous firing. Experimental disambiguation of these dynamical system states has therefore been challenging.

## Discussion

In this study, we provide evidence that regulation of electrophysiology can occur in a low dimensional projection of a neuron’s space of possible ion channel modulations. The implications of this finding for the design of future experiments and targets for therapeutic modulation of the nervous system are several. Furthermore, our ability to replicate, using population modeling, optimization and regression, many key findings from two decades of research by multiple labs is noteworthy. We used a novel, population-based evolutionary algorithm to discover continuous regions of parameter space replete with good models and spanning ranges of target features. Leveraging observations of STOs in recordings from adult WT rodent SNc DAs to constrain the relationships between subthreshold current parameters, our unbiased search found a region of parameter space in which the full range of STO features can be reproduced within a low dimensional, linear control plane. This plane can be used as a basis for discovering a population of models with spiking features covering most of the range described in a set of empirical observations, which generalize to recreate the effects of several experimental perturbations and describe potential regulators of DA activity.

### Electrophysiological control by colinear combinations of intracellular parameters

Our optimization discovered parameter space regions in which models spread across the range of acceptable outputs, from a naive starting point. Prior optimization approaches used a single ideal feature set as a target [48–51, 53, 55, 74], and database approaches targeted uniformity in parameter space but not feature space [31, 75]. Related work in cardiac myocyte [12, 59, 76] and neuron modeling [13] achieved parameter sensitivity analysis by varying parameters around an existing, already established seed. These approaches did not utilize unbiased search to discover the model population as reported here. Recent advances in cardiac modeling have focused on how to find parameter sets for models that reflect the diversity present across cellular populations [77], including the use of distribution fitting to adapt model databases to experimental feature distributions, demonstrating enhanced prediction of perturbation response in the realistically distributed population [78]. Here we fit to feature ranges and encourage an even sampling of a uniform feature distribution, but the resulting model population could be further sampled to achieve a fit to specific data sets by applying distribution fitting in future.

It is known that ion channel properties are intrinsically variable, and yet correlated to preserve function [22]. Studies have showed that overexpression of one channel can lead to a compensatory enhancement of an opposing current [25, 30], and demonstrated computationally that a single intracellular property (e.g. calcium concentration) governing expression of multiple ion channels can result in functional homeostasis, maintaining activity in response to a perturbation by simultaneously adapting several currents [79]. Additionally, similar activity patterns can result from variable conductance levels [80], and pharmacological blockade of one current can produce different activity from neurons that were at different balance points [57]. Examples of this principle can be found in the results of our parameter search, with neuron models within the target range of features produced by different combinations of parameters, leading to different responses to simulated channel blockade (Figs 5 and 6). Our study accounts for feature variability by linearly varying combinations of kinetic parameters, mirroring evidence from weakly electric fish electrocytes, which showed coordination between the control of electric organ discharge frequency and ion channel kinetics [29].

Within the model population we observed a relatively weak correlation of 0.34 between STO and firing frequencies (Fig 4B). Despite our then constraining parameters for channels not blocked by TTX and TEA to the plane of STO control, the STO frequency-predicting coefficient had only a moderate influence on the firing frequency (Fig 4C). We then observed, through simulated instantaneous TTX and TEA washout, that ion channels occupy fundamentally different states in the two regimes even when frequencies align (Fig 7). We therefore conclude that although the same channels play a role in generating and influencing both STO and pacemaking frequencies, these phenomena are not trivially connected, aligning our study with prior modeling work [18]. We arrived at this model population using unbiased evolutionary search conducted from a naive starting point within empirically observed parameter ranges, using features of the STO as clues that the search could follow to achieve realistic balance of ion channel parameters. This confirmation of prior modeling conclusions, and the generalizations of the models to channel blockade (Figs 5 and 6), were not *a priori* constraints on the search, and therefore indicate the potential of the resulting model population to generate new predictions of currently unknown properties of SNc DAs, such as the precise influence of intracellular parameters upon electrophysiological features described herein (Figs 3B, 4C, 4D and 6D and S4 Fig).

Modeling has previously demonstrated that different plausible firing patterns that relate to different DA subpopulations can be generated by varying SK conductance, with lower activation of SK co-occurring with increased bursting activity [8]. Furthermore, [36] showed that burst firing is a hallmark of juvenile SNc DAs and the transition from P2 to P8 is likely mediated by increased SK current. Here we imposed a constraint on spontaneous firing regularity and prevented spontaneously bursting models from emerging. A subset of our final population nevertheless entered bursting regimes under SK blockade (Fig 6). As such, this SNc DA model is capable of producing bursting activity, and we expect that given the correct target features during optimization, multiple DA model populations with various activity modes could be attained, mirroring developmental stages or adult subpopulations.

Our results point to a naive, optimal, and unbiased means to identify which channel currents can be targeted by drugs or intracellular processes to efficiently regulate neuronal properties around the population mode. If, for example, a DA experiences a persistent change in input activity that reduces the frequency of dendritic calcium influx and [Ca^2+^], feedback modulation targeting those ion channels along the FPC’s parameters (Fig 3) could efficiently restore prior function. Previously, [79] implemented a feedback model of such functional homeostasis within a crab stomatogastric ganglion neuron model, using a single, activity-dependent intracellular process ([Ca^2+^]) that governed expression of multiple ion channels. Our method discovers optimal ratios of channel properties and their transformations required to implement this feedback control.

### Specific predictions of DA properties

The parameter coefficients found here align with prior experimental and modeling work on the SNc DA STO. In the influential model of [17], dendritic diameter sets STO frequency because diameter dictates the rate of change of [Ca^2+^]. In our results diameter was fixed but ${\bar{g}}_{\text{CaL}}$ and *V*_half,CaL_ consistently modulated STO frequency (Figs 2G and 3B). Other parameters with strong influence on STO frequency were ${\bar{g}}_{\text{KERG}}$ and *V*_half,KERG_, and previous models showed that blockade of KERG increases STO frequency [81]. Our method uncovered this relationship and the specific ratio between KERG and CaL currents controlling frequency is predicted.

Our final population of SNc DA models had a significant correlation coefficient of 0.34 between STO and firing frequencies. All parameter sets found produced outputs matching SNc DA electrophysiology, and proved robust to ion channel perturbations, suggesting that all are viable SNc DA models. As such, we can consider the population a potential surrogate for the real SNc DA population and therefore capable of yielding new insights into this important neuron type. The positive correlation predicted between STO and firing frequencies is supported by one piece of evidence [19], but further data could confirm whether the relationship and distributions of our model population are accurate.

Our population approach allowed us to produce variable responses to drug-like, channel blockade perturbations across hundreds of parameter sets for the same model formulation (Figs 5 and 6). Only a subset of models responded to SK blockade with CV ISI increase, replicating experimental observations [32] and leading to the prediction that only SNc DAs with lower Kv2 conductance are susceptible to bursting under SK blockade. This type of prediction is impossible to formulate from a single parameter set representing an average neuron. In such a case, we may have blocked SK and reduced Kv2 conductance, only to find that CV ISI remained low. Of course, the model would still be plausible because both low and high CV ISI under SK block are realistic responses among SNc DAs.

Specifically, we predict for the entire population of SNc DAs: 1. the ratios for ion channel modulation which control STO frequency and amplitude (e.g., the ratio of KERG and CaL currents capable of controlling STO frequency; Fig 3B); 2. a correlation of 0.34 between STO and firing frequencies (Fig 4B); 3. the ratios of ion channel modulation which control pacemaking frequency (Fig 4C) 4. a relatively low Kv2 conductance for burst induction under SK block (Fig 6C); and 5. the ratios of NaT, Kv2 and BK currents which best predict ISI CV under SK block (Fig 6D).

### Low-dimensional regulation of intracellular function discovered in a projection of the non-linear model

Our novel method discovered how a high-dimensional, experimentally observed system of interacting ion channels might be controlled. Subthreshold oscillatory activity was captured thousands of times by varying parameters independently over a broad range. By asking how simpler modulations of these parameter sets might reproduce the full range of natural oscillations, we discovered a two dimensional projection of the full parameter space sufficient to control the system. The PCs that were found to be most correlated with subthreshold features across multiple trials were always those accounting for a low amount of the variance among parameter sets, and the precise reason for this remains an open question.

One limitation of the current study derives from the lack of detailed experimental characterization of SNc DA STO. While high voltage STO amplitude was characterized in [57], frequency was not. We assumed a frequency similar to the spontaneous FR reported in [57], which may be incorrect, despite [82] reporting a similar though slower frequency of STO under TTX compared with pacemaking. Furthermore, under blockade of potassium channels with TEA, some SNc DAs enter a mode of high-threshold calcium spiking that can be resilient to apamin [15, 57]. With more precise feature values, our optimization approach might be used to identify parameter combinations necessary to regulate each of these modes.

When utilizing this low-dimensional projection of subthreshold parameter space as a foundation from which to extend the parameter search into the space of parameters for spiking currents, our optimization was unable to sample the full range of some features, most notably the delay to first spike after hyperpolarization, which was at the low end of the empirically observed range for all models found (Fig 4A). In the **Results** section, we suggested that this is possibly due to the low-dimensional subspace overly constraining KA channel kinetic parameters, which are known to be correlated with this feature in the empirical population [4]. To test such constraints, the optimization could be continued from the current population of models, but with only the most constrained feature included in the crowdedness function, encouraging the development of a model population by the algorithm that spreads maximally in this feature. Note that all other constraints would still be included in the error function, ensuring the quality of newly generated models. If regions of the feature space were still inaccessible, then the optimization could be continued with all parameters free. Although the high-dimensional optimization was too complex to find any good models from a naive start, starting from a population of known good models and observing the spread in parameter space that maintains all feature constraints would be a method of testing if the low-dimensional subspace overly constrains parameters. If models with longer delays to first spike could be found using this approach, then a linear subspace precludes discovery of those good models. We gained greater prediction accuracy using a 296-parameter, second order non-linear fit (see S3 Fig), and the possibility that certain non-linear fitting may facilitate dimensionality reduction during parameter search that facilitates recovery of a larger region of feature space than strictly linear reductions represents an important direction for future work.

This study has produced hundreds of potential neuron models, each with a unique combination of intracellular parameters, and each equally valid according to our empirically derived criteria. The model database resulting from our parameter search provides a unique resource for exploring the relationship between ion channel properties and activity patterns among SNc DAs. Future work can take several directions. These optimization techniques can be used with alternate feature ranges to generate model subpopulations representative of, for example, the developmental stages described in [36], or subpopulations defined by anatomy or function within the midbrain, potentially extending to the striking differences between mesoaccumbal and nigrostriatal DAs [4]. Exploration of the role of additional sources in feature variation, such as morphology [52], can be addressed by future optimizations using the same algorithm, possibly couped to parametric models of neurogensis [83]. This database also offers the opportunity to explore population responses to drugs or drug combinations within this neuron type.

### Conclusion

We discovered low-dimensional controllers that provide a simple description of high-dimensional parameter changes that consistently modulate electrophysiological features. Our combination of optimization and analysis provides a powerful tool for uncovering key regulatory constraints and relationships among many intracellular mechanisms [33], and presents numerous testable hypotheses while suggesting the possibility that our low dimensional projections can discover nature’s real canvas upon which functional regulation of neurons and neural systems is composed.

## Supporting information

S1 Methods Ion channel tuning procedures. Details of how baseline parameters for each ion channel model were matched to experimental observations of currents in rodent SNc DAs.
(PDF)

S1 Fig PCA on subthreshold parameter space. (A) PCA: variance explained (% total variance among parameter sets) by each PC. (B) Histogram of scores for the model population for 4 of the PCs. Red lines represent best fit Gaussian distribution centered at 0. (C) An axis of robustness in parameter space was discovered by performing PCA independently on nine populations of models, separated by dividing feature space into a uniform three by three grid according to STO frequency and amplitude. Bar plots show the first PC from independent PCA on parameters from the 4 corner groups of models. The major axis of variance through parameter space is always identical, regardless of location of model in feature space, indicating that parameters must be maintained according to this ratio for a model to be discovered as a ‘good’ model.
(PDF)

S2 Fig Second order polynomial regression improves prediction accuracy for both STO frequency and amplitude. First-order and second-order fits of parameters to features using polynomial regression (Matlab package MultiPolyRegress (https://github.com/ahmetcecen/MultiPolyRegress-MatlabCentral)). Plots show actual feature value (y-axis) and feature value predicted by each parameter set according to the fix (x-axis). A) Linear fit to STO frequency. B) Linear fit to STO amplitude. C) Second order polynomial fit to STO frequency. D) Second order polynomial fit to STO amplitude.
(PDF)

S3 Fig Spontaneous firing model parameter space. Histograms and pairwise comparison scatter plots of parameter values for all 727 good models from the final population of the spontaneous firing optimization.
(PDF)

S4 Fig Spontaneous firing feature regression coefficients. Feature-predicting coefficients from conducting PLSR on all 727 good models from the final population of the spontaneous firing optimization, for spiking features.
(PDF)

S5 Fig Feature calculation. Schematic illustration of feature calculations to capture features listed in Table 1.
(PDF)
