# Supplementary material for: Dimensions of control for subthreshold oscillations and spontaneous firing in dopamine neurons
Source: PLoS Comput Biol. 2019 Sep 23;15(9):e1007375. doi: 10.1371/journal.pcbi.1007375 (PMC6776370; doi:10.1371/journal.pcbi.1007375)
Supplement: S1 Methods — Details of how baseline parameters for each ion channel model were matched to experimental observations of currents in rodent SNc DAs. (PDF) [file pcbi.1007375.s001.pdf]

Dimensions of control for subthreshold oscillations and spontaneous firing in dopamine neurons - Supporting Methods

Timothy Rumbell[1*] James Kozloski[1]

1 IBM Research, Computational Biology Center, Thomas J. Watson Research Laboratories, 1101 Kitchawan Road, Yorktown Heights, NY 10598, USA.

* thrumbel@us.ibm.com

S1 Methods

Ion channels

Most channel models used the Boltzmann formulation for voltage-dependent gating of activation and/or inactivation:

$$x_{\mathrm{inf}}(V) = 1/(1 + (exp(-(V - V_{\mathrm{h}})/k))), \tag{1}$$

where $x_{\mathrm{inf}}(V)$ represents the steady state value of the gating variable x at membrane potential V, V_{h} is the half activation/inactivation voltage, and k is the slope of the activation/inactivation curve.

Most gating variables in our models follow the update equation:

$$\frac{dx}{dt} = -[x - x_{\mathrm{inf}}(V)]/\tau_x(V), \tag{2}$$

where $\tau_x(V)$ is the time constant of decay of the gating variable to steady state.

For each ion channel we introduced meta-parameters V_{half} and τ_{mod} to adjust the half-activation voltage and time constants in accordance with any reported variation in these properties among SNc DAs. We therefore used modified equations for 3 and 4, which incorporate these adjustments:

$$x_{\text{inf}}(V) = 1/(1 + (exp(-V - V_{\text{half}})/k)), \tag{3}$$

and

$$\frac{dx}{dt} = -[x - x_{\text{inf}}(V)]/[\tau_{\text{mod}} \times \tau_x(V)]. \tag{4}$$

Time constants were also subject to a temperature-dependent scaling using a Q10 rule where $Q10$ and *temp* parameters are described for a channel model.

Ion channel tuning procedures

Here we outline the tuning procedure for the equations underlying gating of each ion channel model. As a basis for each we used existing channel models that have been incorporated into previously published single cell models of SNc DAs. In general, parameter tuning was performed on ion channel models that had been derived from neuron types other than SNc DAs, and aimed to ensure that activity of the channels resembled recordings that were performed in SNc DAs. This section provides a detailed summary of the parameter tuning and exact parameter values of the model used for each channel type.

Transient sodium channel (Nav1, NaT) NaT channels are voltage-gated sodium channels that contribute the primary depolarizing drive during the action potential. In SNc DAs, they also may produce a residual current at subthreshold voltages, contributing to the depolarizing drive leading to *in vitro* pacemaker firing [19]. We used an established SNc DA NaT model, introduced in [84] to match SNc DA recordings [85] and refined in [72] to capture the slow component of inactivation seen in this channel in this neuronal population [86]. The channel model was as follows:

$$g_{\mathrm{NaT}} = \bar{g}_{\mathrm{NaT}} m^3 h_{\mathrm{f}} h_{\mathrm{s}}), \tag{5}$$

where m, h_{f} and h_{s} are steady state gating variables updated according to equation 3, with time constants τ in equation 4 updated according to:

$$\tau_{\mathrm{m}}(V) = t_{0,\mathrm{m}} + 1/(\mathrm{a}+\mathrm{b}), \tag{6}$$

$$\mathrm{a} = Ct_{\mathrm{m}}(at_{0,\mathrm{m}} + (at_{1,\mathrm{m}}V))/(exp(at_{0,\mathrm{m}} + (at_{1,\mathrm{m}}V)) - 1), \tag{7}$$

$$\mathrm{b} = bt_{0,\mathrm{m}} * exp(bt_{1,\mathrm{m}}V), \tag{8}$$

$$\tau_{h_{\mathrm{f}}}(V) = t_{0,\mathrm{h}} + 1/((at_{0,\mathrm{h}}exp(at_{1,\mathrm{h}}V)) + (bt_{0,\mathrm{h}}exp(bt_{1,\mathrm{h}}V))), \tag{9}$$

$$\tau_{h_{\mathrm{s}}}(V) = t_{0,\mathrm{hs}} + t_{1,\mathrm{hs}}/(1+exp(V)). \tag{10}$$

Additional parameters were incorporated during the optimization to allow adjustment of the voltage dependence of each inactivation component relative to the voltage dependence of activation. The parameter $vh_{\mathrm{h,shift}}$ and $vh_{\mathrm{hs,shift}}$ were applied as modifiers to the vh_{h} and vh_{hs}, respectively, in equation 3. We also included parameters to alter the NaT channel in the AIS segment, multiplying the $\bar{g}_{\mathrm{NaT}}$ parameter used in all other compartments by a free parameter $g_{\mathrm{ax,NaT}}$, and adjusting the vh_{m}, vh_{h} and vh_{hs} values used in all other compartments by a free parameter $vh_{\mathrm{ax,NaT}}$.

Hyperpolarization-activated cyclic nucleotide-gated cation channel (HCN)

HCN channels are voltage-gated, hyperpolarization-activated, non-selective cation channels that produce a pronounced depolarizing current in response to membrane hyperpolarization. Many *ex vivo* studies [66, 82, 87] implicated HCN channels in various SNc DA phenomena, such as prominent 'sag' membrane responses during prolonged hyperpolarizing current step injections. *In vivo*, HCN channels' vital role may include tuning SNc DAs responses to strong GABAergic inputs received *in vivo* [26].

Another important property of HCN channels is their gating dependence on cyclic adenosine monophosphate (cAMP), which facilitates channel activation upon binding by shifting the channel's effective voltage dependence. The HCN channel model of [88]

Parameter	Value	Unit
vh_{m}	-30.0907	mV
k_{m}	9.7264	mV
$t_{0,\mathrm{m}}$	0.01	ms
Ct_{m}	0.79992	ms
$at_{0,\mathrm{m}}$	-19.565	mV
$at_{1,\mathrm{m}}$	-0.50542	mV
$bt_{0,\mathrm{m}}$	3.0212	mV
$bt_{1,\mathrm{m}}$	-0.007463	mV
vh_{h}	-54.0289	mV
k_{h}	-10.7665	mV
$t_{0,\mathrm{h}}$	0.4	ms
$at_{0,\mathrm{h}}$	0.00050754	mV
$at_{1,\mathrm{h}}$	-0.063213	mV
$bt_{0,\mathrm{h}}$	9.7529	mV
$bt_{1,\mathrm{h}}$	0.13442	mV
vh_{hs}	-54.8	mV
k_{hs}	-1.57	mV
$t_{0,\mathrm{hs}}$	20	ms
$t_{1,\mathrm{hs}}$	580	ms
$Q10$	2.3	
$temp$	23	C

Table 1. Tuned NaT parameters used in m, h_{f} and h_{s} components of equation 5, according to default formulation in equation 3 for steady state values of m and h components, and equation 6 for time constants. h_{s} component has no time constant voltage dependencies. Only activation depends on voltage.

incorporates this effect, and has therefore been used in various SNc DA modeling studies [68, 89]. We chose to use this HCN channel model as a starting point for incorporating this important current into our model. The channel model was formulated as follows:

$$g_{\mathrm{HCN}} = \bar{g}_{\mathrm{HCN}}(O + AO), \tag{11}$$

where O and AO are the open and open, cAMP-bound gating states of the channel, respectively. These are updated, along with the closed (C) and closed, cAMP-bound (AC) states, according to the Markov model illustrated, with rate functions as in equation 12.

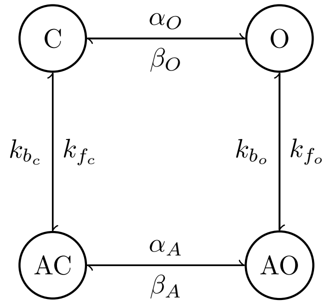

Markov process for HCN channel gating

$$\alpha_O = a_0/1 + exp(-(V - ah) * ac) \tag{12}$$

$$\beta_O = b_0/1 + exp(-(V - bh) * bc) \tag{13}$$

$$\alpha_{\mathrm{A}} = aA_0/1 + exp(-(V - aAh) * aAc) \tag{14}$$

$$\beta_{\mathrm{a}} = bA_0/1 + exp(-(V - bAh) * bAc) \tag{15}$$

$$k_{f_c} = k_{\mathrm{on}} ai \tag{16}$$

$$k_{b_c} = k_{\mathrm{off}} \tag{17}$$

$$k_{f_o} = k_{\mathrm{on}} \frac{ai}{bf} \tag{18}$$

$$k_{b_o} = k_{\mathrm{off}} \frac{b}{bf} \tag{19}$$

We adjusted the kinetic parameters of these rate functions to better match observations from voltage clamp recordings of HCN channels in SNc DAs [24, 90]. In these studies, HCN shows more depolarized activation relative to the default model of [88], which was originally parameterized for a thalamocortical relay neuron simulation. We used the same cAMP binding rates as in the original formulation, but allowed forward and backward activation rate parameters to vary, tuning the voltage dependence of activation and time constants of the model to approximate the mean of empirical recordings reported in [90] and [24] for each of the cAMP bound, partially bound, and unbound states.

The data we used to choose adjustment parameters V_{half} and τ_{mod} came from Table 1 and Table 2 of [24]. As equations 3 and 4 were not used in this case, V_{half} and τ_{mod} instead acted on combinations of gating variable parameters, as in Table 2. Based on

Parameter	Value	Unit
a_0	0.00032743	ms^{-1}
ah	$-87.7 + vh_{\mathrm{shift}}$	mV
ac	-0.1103	mV^{-1}
b_0	0.00029334	ms^{-1}
bh	$-51.7 + vh_{\mathrm{shift}}$	mV
bc	0.1025	mV^{-1}
aA_0	0.0011	ms^{-1}
aAh	$-94.2 + avh_{\mathrm{shift}}$	mV
aAc	-0.0774	mV^{-1}
bA_0	0.0164	ms^{-1}
bAh	$-35.5 + avh_{\mathrm{shift}}$	mV
bAc	0.1486	mV^{-1}
k_{on}	3.086	$\mathrm{mmol}^{-1}\,\mathrm{ms}^{-1}$
ai	1×10^{-5}	mmol
k_{off}	4.4857×10^{-5}	ms^{-1}
bf	8.94	a.u.
b	80	a.u.
vh_1	1.057	a.u.
vh_2	79.3	mV
avh_1	1.886	a.u.
avh_2	164.1	mV
vh_{shift}	$V_{\mathrm{half}} \times vh_1 + vh_2$	mV
avh_{shift}	$V_{\mathrm{half}} \times avh_1 + avh_2$	mV

Table 2. Tuned HCN parameters used in equation 12.

these, we first assumed that shifts in the voltage dependence of activation of 3 mV and 10 mV should be observed in response to cAMP concentrations of 0.000 01 mmol and 0.05 mmol, relative to the half activation in absence of cAMP. We then tuned parameters using least squares non-linear regression so that the activation and time constant curves matched all cAMP levels simultaneously. Next, we used the additional parameters vh_1, vh_2, avh_1 and avh_2 to adjust several model parameters determining the half activation voltage $V_{\mathrm{half,HCN}}$, in accordance with observations of [24] and [90] that half activation voltage of HCN channels in SNc DA varies over a wide range. We set these additional parameters by performing the least squares fitting process with 26 different values of v_{h} in the target Boltzmann equation (according to the observed range of half-activation values from [24]) and assessing which model parameters needed adjustment in order to consistently shift the half-activation voltage of the model. This approach enabled exploration of how varying the voltage dependence of this crucial channel affects our subthreshold optimization. Table 2 shows the resulting tuned values for the model parameters.

T-type calcium (Cav3) T-type calcium channels are voltage-gated calcium channels, with characteristic low voltage dependence of activation and rapid inactivation [91]. Cav3.1 and Cav1.3 channels in SNc DAs are inversely coregulated, such that up regulation of Cav3.1 mRNA expression coincides with the absence of Cav1.3 [92]. Inverse coregulation suggests flexibility in the comodulation of SNc DA activity patterns and in the control of calcium-dependent mechanisms. Multiple sources of calcium influx play a role in activation of SK in SNc DAs [32]. There is evidence from juvenile animals that calcium influx through T-type channels is prominently involved in SK activation and that Cav3.1 can thereby directly regulate SK to provide stability to pacemaker firing *in vitro* and prevent intrinsic bursting [93].

In neonatal rats, SK activation can result from spontaneous single T-type calcium channel opening paired with amplification of calcium influx via internal calcium release through ryanodine receptors. This observation led [94] to conclude that Cav3.1 also modulates firing regularity. In addition, dendritic calcium transients in SNc DAs result from a hyperpolarization induced afterdepolarization, which could play an influential, unique role in the response properties of SNc DAs. These transients are mediated by Cav3 channels [95], and interestingly, channel labeling studies of Cav3.1 show expression in SNc DAs restricted to the soma early in development, but then emerging later among dendrites [96].

Voltage clamp recordings of Cav3 channel inactivation in SNc DAs are fit better by models that include fast and slow components of inactivation [92], which we therefore include in our model. The channel model was formulated as follows:

$$g_{\mathrm{Cav3}} = \bar{g}_{\mathrm{Cav3}} m^3 (0.6 h_{\mathrm{f}} + 0.4 h_{\mathrm{s}}), \tag{20}$$

where m, h_{f} and h_{s} are steady state gating variables updated according to equation 3, with time constants τ in equation 4 updated according to:

$$\tau_{\mathrm{m}}(V) = t_{0,\mathrm{m}} + Ct_{\mathrm{m}}/(1 + exp((V - vht_{\mathrm{m}})/at_{\mathrm{m}})), \tag{21}$$

$$\tau_{h_{\mathrm{f}}}(V) = t_{0,\mathrm{h}} + Ct_{\mathrm{h}}/(1 + exp((V - vht_{\mathrm{h}})/at_{\mathrm{h}})), \tag{22}$$

$$\tau_{h_{\mathrm{s}}}(V) = t_{0,h_s}. \tag{23}$$

Parameters in the kinetic equations 3 and 21 were tuned to approximate observed voltage dependence of activation and time constants [92]. We began the tuning process with parameters defined by [91] for the α1H subunit, which appeared to more closely match the time constants found in the SNc DAs than the α1G subunit (Cav3.1 and Cav3.2 mRNAs are both observed in SNc DAs [92]). For tuning, we fixed parameters for voltage dependence of activation at $vh_{\mathrm{m}} = -45$ mV, leading to a half-permeability value close to the observed value of -40.1 [92] when raised to the power of 3 (a power of 3 m gate was used in the VTA DA neuron model of [4]). We also fixed $k_{\mathrm{m}} = 3.6$ mV based on the permeability curve of [92]. Neuron's Multiple Run Fitter's praxis algorithm was used to tune the current produced by the CaT channel model by altering all parameters in equation 21 to approximate the mean time-to-peak and sum of two exponentials decay in the empirical data at 4 different voltage clamps, stepping from -100 mV to -50, -40, -30 and 0 mV. Target time-to-peak-currents used at each of these voltage steps were 62.1, 50.6, 39.1 and 26.0 ms respectively. Target fast time constants of inactivation used in the sum of two exponentials equation were 152.1, 105.9, 86.3 and 75.1 ms for each voltage step respectively. The targeted slow time constant of inactivation was 661.3 ms and voltage independent for all voltage steps. The fast component of inactivation accounted for 60% of the sum [92, 97].

With kinetic parameters of the channel model set to allow current time courses to approximate observations (see Table 3 for parameters), we next added $V_{\mathrm{half,CaT}}$ and $\tau_{\mathrm{mod,CaT}}$ variables to the channel model so as to control and shift the voltage dependence and magnitude of all time constants in accordance with the range of properties observed across the data and SNc DA population. For this channel, $V_{\mathrm{half,CaT}}$ shifted both m and h_f, to maintain the 'window' current between activation and inactivation. We included three parameters in the subthreshold optimization, $\bar{g}_{\mathrm{CaT}}$, $V_{\mathrm{half,CaT}}$ and $\tau_{\mathrm{mod,CaT}}$, used in equations 3, 4, and 20.

Parameter	Value	Unit
vh_{m}	-54.5	mV
k_{m}	5	mV
$t_{0,\mathrm{m}}$	3.2	ms
Ct_{m}	19	ms
vht_{m}	-40	mV
at_{m}	4.6	mV
vh_{h}	-64.5	mV
k_{h}	-1.6	mV
$t_{0,\mathrm{h}}$	76	ms
Ct_{h}	43	ms
vht_{h}	-46	mV
at_{h}	8.85	mV
vh_{hs}	-64.5	mV
k_{hs}	-1.6	mV
$t_{0,\mathrm{hs}}$	600	ms
$Q10$	3.0	
$temp$	33	C

Table 3. Tuned Cav3.1 parameters used in m, h_{f} and h_{s} components of equation 20, according to default formulation in equation 3 for steady state values of m and h components, and equation 21 for time constants. h_{s} component has no time constant voltage dependencies. Only activation depends on voltage.

L-type calcium (Cav1.3) L-type calcium channels are important in determining subthreshold dynamics in DAs. They contribute greatly to the calcium current [98] in proportions that change gradually throughout development [68]. In DAs, L-type calcium channels are commonly reported as Cav1.3 [99], the low-voltage-gated form of the L-type calcium channel [100]. This implicates L-type calcium currents in regulating subthreshold membrane potential and enables currents from L-type channels to contribute to large Ca^{2+} transients at subthreshold potentials in DA soma and dendrites [17]. Additionally, activation of SK currents through calcium influx via calcium channels leads to regularization of spike timing, with L-type calcium implicated [32]. Cav1.3 channels are also subject to calcium-dependent inactivation (CDI) and calcium-dependent facilitation (CDF), each caused by Ca^{2+} influx due to Cav1.3 channel activation [101].

The hyperpolarized activation of L-type channels has been emphasized in DA models, with some models incorporating half-activation voltages at -45 mV or lower [18, 21, 102, 103, 105]. Evidence instead suggests a half-activation above -30 mV for these channels [98, 99]. We therefore implemented a Cav1.3 model influenced by the activation and partial inactivation kinetics and activation time constant variability

of [99] (in this study, the use of 20 mM Ba^{2+} as charge carrier, as opposed to ∼2 mM Ca^{2+}, led to a rightward shift in voltage dependence of steady state activation and inactivation [106]).

We used a voltage dependence curve for activation derived from the data of [98] and included in our model their reported half-activation variability as well as the difference between voltage-dependent activation (VDA) and inactivation (VDI) reported in [100]. With these, we determined a difference in half-activation and half-inactivation voltages, as established in [107]. [99] and [100] each showed that inactivation maximally blocks about 80% of the current, so we used 80% as an upper limit for VDI and rescaled the h variable calculated from equation 3 to between 0 and 0.8.

Furthermore, activation is fast in Cav1.3 L-type channels [107], so we modified the equation of [69], which approximates voltage-dependence of activation time constant data from [108], according to:

$$g_{\mathrm{Cav1.3}} = \bar{g}_{\mathrm{Cav1.3}} m^2 h f, \tag{24}$$

where the m (VDA) and h (VDI) variables were updated according to equation 3. CDI (f) was updated according to:

$$f_{\mathrm{inf}} = 1/(1 + ([Ca^{2+}]_i/kf)), \tag{25}$$

and time constants for m, h and f, used in equation 4, were updated according to:

$$\tau_{\mathrm{m}}(V) = t_{0,\mathrm{m}} + Ct_{\mathrm{m}}/(1 + exp((V - vht_{\mathrm{m}})/at_{\mathrm{m}})), \tag{26}$$

$$\tau_h(V) = t_{0,\mathrm{h}} + Ct_{\mathrm{h}}/(1 + exp((V - vht_{\mathrm{h}})/at_{\mathrm{h}})), \tag{27}$$

$$\tau_f = 30. \tag{28}$$

Using this form, we scaled time constants to approximate the data of [99], recorded from SNc DAs. The resulting parameters are shown in Table 4. VDI in L-type channels

Parameter	Value	Unit
vh_{m}	−35	mV
k_{m}	5.5	mV
$t_{0,\mathrm{m}}$	0.2	ms
Ct_{m}	0.2	ms
vht_{m}	−35	mV
at_{m}	12	mV
vh_{h}	−56	mV
k_{h}	−8	mV
$t_{0,\mathrm{h}}$	300	ms
kf	0.01	nM
τ_f	30	1/ms
$Q10$	2.0	
$temp$	30	C

Table 4. Tuned Cav1.3 parameters used in m, h and f components of equation 24, according to the default formulation of equation 3 for m and h steady state voltage dependence, equation 25 for CDI, and equation 26 for time constants.

is usually considered slow relative to both VDA and CDI, and in the absence of more precise data, we chose an approximate average value of 300 ms from a survey of multiple studies of various neurons [107]. We then varied this property proportional to the time constant of VDA during our optimizations.

Data for the CDI is also scarce, so we used the value of 0.01 nmol from [103] as the parameter kf (equation 8 of [107]), leaving this parameter free so that the model could adapt to the new calcium dynamics derived by the optimization. We set parameter τ_f from the same equation to its default value of 30 ms, as in [107], providing fast CDI characteristics relative to VDI, and then scaled this time constant with the VDA and VDI time constants during the optimization. Four parameters were then included in the subthreshold optimization, $\bar{g}_{\mathrm{CaL}}$, $V_{\mathrm{half,CaL}}$, $\tau_{\mathrm{mod,CaL}}$, and kf_{CaL}, used in equations 3, 4, 25, and 26, thus allowing L-type calcium currents to vary according to the range of observations from SNc DAs. For this channel, $V_{\mathrm{half,CaL}}$ shifted both m and h, to maintain the 'window' current between activation and inactivation.

Delayed rectifier potassium (Kv2, KDR) Rectifying, voltage-dependent, Kv2 potassium channels contribute substantially to action potential repolarization in SNc DA neurons [64, 96]. We matched kinetics to those described in [64], recorded from mouse SNc DA neurons, modeled using the following:

Parameter	Value	Unit
vh_{m}	-30.5	mV
k_{m}	-13	mV
$t_{0,\mathrm{m}}$	3.6	ms
Ct_{m}	3.6	ms
vht_{m}	-9	mV
at_{m}	10	mV
$Q10$	3	
$temp$	34	C

Table 5. Kv2 parameters used in the m component of equation 29, according to default formulation in equation 3 for m steady state voltage dependence and equation 30 for time constants.

$$g_{\mathrm{Kv2}} = \bar{g}_{\mathrm{Kv2}} m^4, \tag{29}$$

where m was updated according to equation 4, with steady state activation according to equation 3. Time constants were updated according to:

$$\tau_m(V) = t_{0,\mathrm{m}} + Ct_{\mathrm{m}}/(1 + exp((V - vht_{\mathrm{m}})/at_{\mathrm{m}})), \tag{30}$$

with baseline parameter values from [64], shown in Table 5. Four parameters were included in the optimization for this current, three of which were $\bar{g}_{\mathrm{Kv2}}$, $V_{\mathrm{half,Kv2}}$, and $\tau_{\mathrm{mod,Kv2}}$, used in equations 3, 4, and 29. The fourth free parameter was a scaling factor, $g_{\mathrm{ax,Kv2}}$, used to scale $\bar{g}_{\mathrm{Kv2}}$ in the AIS, relative to the parameter value selected for $\bar{g}_{\mathrm{Kv2}}$ in all other compartments, and relative to the scaling factor $g_{\mathrm{ax,NaT}}$ applied to $\bar{g}_{\mathrm{NaT}}$ in the AIS. As such, the value of $\bar{g}_{\mathrm{Kv2}}$ in the AIS was $\bar{g}_{\mathrm{Kv2}}(g_{\mathrm{ax,Kv2}})g_{\mathrm{ax,NaT}}$.

Large-conductance potassium (BK) Another prominent component of action potential repolarization in SNc DA neurons is the large-conductance, voltage-gated, calcium-modulated BK current [64]. This current activates more rapidly than Kv2, leading to a different role in forming action potential shape [64]. Although the voltage-dependence of BK current activation is calcium-modulated [109], and can be more accurately modeled including this modulatory effect [110], here we chose to model only the calcium-independent function of the channel to most simply match the model

Parameter	Value	Unit
vh_{m}	-16	mV
k_{m}	-8.5	mV
$t_{0,\mathrm{m}}$	0.87	ms
Ct_{m}	0.13	ms
vht_{m}	-16	mV
at_{m}	10	mV
$Q10$	3	
$temp$	34	C

Table 6. BK parameters used in the m component of equation 31, according to default formulation in equation 3 for m steady state voltage dependence and equation 32 for time constants.

current to the available data recorded in mouse SNc DAs [64].

The following equations were used:

$$g_{\mathrm{BK}} = \bar{g}_{\mathrm{BK}} m, \tag{31}$$

where m was updated according to equation 4, with steady state activation according to equation 3. Time constants were updated according to:

$$\tau_m(V) = t_{0,\mathrm{m}} + Ct_{\mathrm{m}}/(1 + exp((V - vht_{\mathrm{m}})/at_{\mathrm{m}})), \tag{32}$$

with baseline parameter values from [64], shown in Table 6. Three parameters were included in the optimization for this current, $\bar{g}_{\mathrm{BK}}$, $V_{\mathrm{half,BK}}$, and $\tau_{\mathrm{mod,BK}}$, used in equations 3, 4, and 31.

Calcium-dependent potassium (SK) Small-conductance, calcium-dependent potassium channels are strongly implicated in a variety of DA neuron activity patterns [6]. They are related to firing rate, adaptation [111], and the transition between tonic and burst firing patterns [63, 71]. SK also plays a pivotal role in the *in vitro* subthreshold oscillation, with SK blockade canceling ongoing slow oscillation and causing plateau potentials and subthreshold calcium spiking [57]. SK channels are gated by calcium through an interaction between the pore-forming SK subunits and calmodulin (CaM) [112], although this can be modeled as a calcium-dependence, using

either a Markov model with 4 closed states and 2 open states, and calcium-dependent rate constants dictating state transitions [113], or more commonly, a Hill equation with half-activation calcium concentration around 0.25 µmol and Hill coefficient around 4 [114]. We used the latter approach, with SK conductance updated according to:

$$g_{\mathrm{SK}} = \bar{g}_{\mathrm{SK}} o, \tag{33}$$

where o is gated instantaneously (i.e. time constant of 0 ms) by $[Ca^{2+}]_{\mathrm{i}}$ according to:

$$o = 1/(1 + (km/[\mathrm{Ca}^{2+}]_{\mathrm{i}})^4), \tag{34}$$

which we used in place of equation 3.

The two parameters $\bar{g}_{\mathrm{SK}}$ and km_{SK} used in equations 33 and 34 were then included in the subthreshold optimization, allowing the calcium dependence of the SK channel to vary according to the intracellular calcium dynamics dictated by other model parameters.

A-type potassium (Kv4.3, KA) Transient A-type potassium currents have been found in SNc DAs [115, 116] and shown to comprise the pore-forming alpha-subunits Kv4.3L (long splice variant) and the auxiliary beta-subunits KChip3.1 [9]. These channels increase interspike interval by opposing depolarizing drive towards threshold [9, 117] and crucially regulate characteristics of AP timing, such as delay to first AP upon rebound from hyperpolarization [24]. KA channels are activated and inactivated at relatively hyperpolarized voltages and have very rapid activation, and slower, although still rapid, inactivation and deinactivation [24, 115, 116]. Here we modeled the current using the equation:

$$g_{\mathrm{Kv4.3}} = \bar{g}_{\mathrm{Kv4.3}} m^3 h, \tag{35}$$

Parameter	Value	Unit
vh_{m}	-40	mV
k_{m}	-7	mV
$t_{0,\mathrm{m}}$	1.029	ms
Ct_{m}	4.83	ms
vht_{m}	-56.7	mV
at_{m}	6.22	mV
vh_{h}	-73	mV
k_{h}	4.9	mV
$t_{0,\mathrm{h}}$	39.04	ms
Ct_{h}	78.4	ms
vht_{h}	-68.5	mV
at_{h}	5.95	mV
$Q10$	23.0	
$temp$	32	C

Table 7. Kv4.3 parameters used in m and h components of equation 35, according to default formulation in equation 3 for m and h steady state voltage dependence and equation 36 for time constants.

where m and h were updated according to equation 4, with steady state activation according to equation 3. Time constants were updated according to:

$$\tau_m(V) = t_{0,\mathrm{m}} + Ct_{\mathrm{m}}/(1 + exp((V - vht_{\mathrm{m}})/at_{\mathrm{m}})), \text{and} \tag{36}$$

$$\tau_h(V) = t_{0,\mathrm{h}} + Ct_{\mathrm{h}}/(1 + exp((V - vht_{\mathrm{h}})/at_{\mathrm{h}})), \tag{37}$$

with baseline parameter values from [24], shown in Table 7, but with a power of 3 for the m gate, as used in [4, 68, 102–104]. Three parameters were included in the optimization, $\bar{g}_{\mathrm{KA}}$, $V_{\mathrm{half,KA}}$, and $\tau_{\mathrm{mod,KA}}$, used in equations 3, 4, and 35, thus facilitating the generation of models with A-type currents that mirrored the full range of values observed in [24].

Ether-a-go-go-related-gene potassium (KERG) A slow, hyperpolarizing current associated with the duration of AP afterhyperpolarization (AHP) has been observed in SNc DAs [16, 118]. This current was characterized by [119], where it was found to be potassium-mediated and have many characteristics in common with the ERG K^+ channel. [81] then demonstrated a haloperidol- (a potent KERG channel blocker) dependent elongation of plateau potentials induced by apamin application in DAs during ongoing subthreshold activity. Modeling of the role of KERG showed that

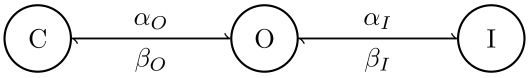

Markov process for gating of KERG current

the slow activation kinetics could lead to termination of the plateaus generated by simulated SK block [81]. [10] showed that KERG can regulate spontaneous firing rate and bursting properties of DAs *in vitro*, and [73] that simulation of the slow components of the KERG channel can play a role in model recovery from depolarization block caused by slow inactivation of the transient sodium current.

We modeled KERG current according to:

$$g_{\mathrm{KERG}} = \bar{g}_{\mathrm{KERG}}\mathrm{O}, \tag{38}$$

where O represents fraction of open channels. Models of KERG channel gating use a three-state kinetic scheme, with transitions between closed (C) and open (O), and between open and inactivated (I) states, according to the Markov process for KERG gating shown.

Voltage-dependent state transition rates α_O, β_O, α_I, β_I were fit in [73] to experimental recordings of the ERG current in Xenopus Oocytes [120, 121], resulting in rates of:

$$\alpha_{\mathrm{O}} = 0.00236\exp(0.0733V), \tag{39}$$

$$\beta_{\mathrm{O}} = 1.2523e^{-05}\exp(-0.0671V), \tag{40}$$

$$\alpha_{\mathrm{I}} = 91.11\exp(0.1189V), \tag{41}$$

$$\beta_{\mathrm{I}} = 12.6\exp(0.0733V). \tag{42}$$

State variables were updated according to:

$$dO/dt = \alpha_O(1 - O - I) + \beta_I(I) - (\alpha_I + \beta_O)(O), \quad (43)$$

$$dI/dt = \alpha_I(O) - \beta_I(I). \quad (44)$$

In the absence of further data for the range of voltage-dependence and time constants of these equations, we introduced parameters $V_{\mathrm{half,KERG}}$ as a voltage shift of up to 5 mV in the de- or hyper-polarized direction applied to the V values in equation 39, and $\tau_{\mathrm{mod,KERG}}$, a scalar multiplier between 0.5 and 1.5 applied to the α and β values in equation 39, to allow KERG current to vary in the approximate range observed for the other currents present in DAs.

References

1. Schultz, W. (1998). Predictive reward signal of dopamine neurons. *Journal of Neurophysiology*, 80(1):1–27.

2. Grace, A. A. (2016). Dysregulation of the dopamine system in the pathophysiology of schizophrenia and depression. *Nature Reviews Neuroscience*, 17:524–532.

3. Khaliq, Z. M. and Bean, B. P. (2008). Dynamic, nonlinear feedback regulation of slow pacemaking by A-type potassium current in ventral tegmental area neurons. *The Journal of Neuroscience*, 28(43):10905–10917.

4. Tarfa, R. A., Evans, R. C., and Khaliq, Z. M. (2017). Enhanced sensitivity to hyperpolarizing inhibition in mesoaccumbal relative to nigrostriatal dopamine neuron subpopulations. *The Journal of Neuroscience*, 37:3311–3330.

5. Neuhoff, H., Neu, A., Liss, B., and Roeper, J. (2002). I_h channels contribute to the different functional properties of identified dopaminergic subpopulations in the midbrain. *The Journal of Neuroscience*, 22(4):1290–1302.

6. Wolfart, J., Neuhoff, H., Franz, O., and Roeper, J. (2001). Differential expression of the small-conductance, calcium-activated potassium channel SK3 is critical for pacemaker control in dopaminergic midbrain neurons. *The Journal of Neuroscience*, 21(10):3443–3456.

7. Vandecasteele, M., Deniau, J.-M., and Venance, L. (2011). Spike frequency adaptation is developmentally regulated in substantia nigra pars compacta dopaminergic neurons. *Neuroscience*, 192:1–10.

8. Oster, A., Faure, P., and Gutkin, B. (2015). Mechanisms for multiple activity modes of VTA dopamine neurons. *Frontiers in Computational Neuroscience*, 9(95):1–17.

9. Liss, B., Franz, O., Sewing, S., Bruns, R., Neuhoff, H., and Roeper, J. (2001). Tuning pacemaker frequency of individual dopaminergic neurons by Kv4.3L and KChip3.1 transcription. *The EMBO Journal*, 20(20):5715–5724.

10. Ji, H., Tucker, K. R., Putzier, I., Huertas, M. A., Horn, J. P., Canavier, C. C., Levitan, E. S., and Shepard, P. D. (2012). Functional characterization of ether-à-go-go-related gene potassium channels in midbrain dopamine neurons – implications for a role in depolarization block. *European Journal of Neuroscience*, 36(May):2906–2916.

11. Evans, R. C., Zhu, M., and Khaliq, Z. M. (2017). Dopamine inhibition differentially controls excitability of substantia nigra dopamine neuron subpopulations through T-type calcium channels. *The Journal of Neuroscience*, 13(37):3704–3720.

12. Sobie, E. A. (2009). Parameter sensitivity analysis in electrophysiological models using multivariable regression. *Biophysical Journal*, 96(4):1264–1274.

13. Taylor, A. L., Goaillard, J.-M., and Marder, E. (2009). How multiple conductances determine electrophysiological properties in a multicompartment model. *The Journal of Neuroscience*, 29(17):5573–5586.

14. Harris, N. C., Webb, C., and Greenfield, S. A. (1989). A possible pacemaker mechanism in pars compacta neurons of the guinea-pig substantia nigra revealed by various ion channel blocking agents. *Neuroscience*, 31(2):355–362.

15. Kang, Y. and Kitai, S. T. (1993b). Calcium spike underlying rhythmic firing in dopaminergic neurons of the rat substantia nigra. *Neuroscience Research*, 18(3):195–207.

16. Nedergaard, S., Flatman, J. A., and Engberg, I. (1993). Nifedipine- and γ-conotoxin-sensitive Ca^{2+} conductances in guinea-pig substantia nigra pars compacta neurones. *Journal of Physiology*, 446:727–747.

17. Wilson, C. J. and Callaway, J. C. (2000). Coupled oscillator model of the dopaminergic neuron of the substantia nigra. *Journal of Neurophysiology*, 83(5):3084–3100.

18. Drion, G., Massotte, L., Sepulchre, R., and Seutin, V. (2011). How modeling can reconcile apparently discrepant experimental results: the case of pacemaking in dopaminergic neurons. *PLoS Computational Biology*, 7(5):e1002050.

19. Guzman, J. N., Sánchez-Padilla, J., Chan, C. S., and Surmeier, D. J. (2009). Robust pacemaking in substantia nigra dopaminergic neurons. *The Journal of Neuroscience*, 29(35):11011–11019.

20. Amini, B., Clark, J. W., and Canavier, C. C. (1999). Calcium dynamics underlying pacemaker-like and burst firing oscillations in midbrain dopaminergic neurons: a computational study. *Journal of Neurophysiology*, 82(5):2249–2261.

21. Kuznetsova, A. Y., Huertas, M. A., Kuznetsov, A. S., Paladini, C. A., and Canavier, C. C. (2010). Regulation of firing frequency in a computational model of a midbrain dopaminergic neuron. *Journal of Computational Neuroscience*, 28:389–403.

22. Golowasch, J. (2014). Ionic current variability and functional stability in the nervous system. *BioScience*, 64(7):570–580.

23. Marder, E. (2011). Variability , compensation , and modulation in neurons and circuits. *Proceedings of the National Academy of Sciences*, 108(suppl. 3):15542–15548.

24. Amendola, J., Woodhouse, A., Martin-Eauclaire, M. F., and Goaillard, J. M. (2012). Ca^{2+}/cAMP-sensitive covariation of I_A and I_H voltage dependences tunes rebound firing in dopaminergic neurons. *The Journal of Neuroscience*, 32(6):2166–2181.

25. Maclean, J. N., Zhang, Y., Goeritz, M. L., Casey, R., Oliva, R., Guckenheimer, J., and Harris-Warrick, R. M. (2005). Activity-independent coregulation of I_A and I_h in rhythmically active neurons. *Journal of Neurophysiology*, 94:3601–3617.

26. Henny, P., Brown, M. T. C., Northrop, A., Faunes, M., Ungless, M. A., Magill, P. J., and Bolam, J. P. (2012). Structural correlates of heterogeneous in vivo activity of midbrain dopaminergic neurons. *Nature Neuroscience*, 15(4):613–619.

27. Meza, R. C., López-Jury, L., Canavier, C. C., and Henny, P. (2018). Role of the axon initial segment in the control of spontaneous frequency of nigral dopaminergic neurons *in vivo*. *The Journal of Neuroscience*, 38(3):733–744.

28. Moubarak, E., Engel, D., Dufour, M. A., Tapia, M., Tell, F., and Goaillard, J.-M. (2019). Robustness to axon initial segment variation is explained by somatodendritic excitability in rat substantia nigra dopaminergic neurons. *The Journal of Neuroscience*, 39(26):5044–5063.

29. McAnelly, M. L. and Zakon, H. H. (2000). Coregulation of voltage-dependent kinetics of Na^+ and K^+ currents in electric organ. *The Journal of Neuroscience*, 20(9):3408–3414.

30. MacLean, J. N., Zhang, Y., Johnson, B. R., and Harris-Warrick, R. M. (2003). Activity-independent homeostasis in rhythmically active neurons. *Neuron*, 37:109–120.

31. Prinz, A. A., Bucher, D., and Marder, E. (2004). Similar network activity from disparate circuit parameters. *Nature Neuroscience*, 7(12):1345–52.

32. de Vrind, V., Scuvée-Moreau, J., Drion, G., Hmaied, C., Philippart, F., Engel, D., and Seutin, V. (2016). Interactions between calcium channels and SK channels in midbrain dopamine neurons and their impact on pacemaker regularity: contrasting roles of N- and L-type channels. *European Journal of Pharmacology*, 788:274–279.

33. Marder, E. and Taylor, A. L. (2011). Multiple models to capture the variability in biological neurons and networks. *Nature Neuroscience*, 14(2):133–138.

34. Gentet, L. J. and Williams, S. R. (2007). Dopamine gates action potential backpropagation in midbrain dopaminergic neurons. *The Journal of Neuroscience*, 27:1892–1901.

35. Engel, D. and Seutin, V. (2015). High dendritic expression of I_{h} in the proximity of the axon origin controls the integrative properties of nigral dopamine neurons. *Journal of Physiology*, 593.22:4905–4922.

36. Dufour, M. A., Woodhouse, A., Amendola, J., and Goaillard, J.-M. (2014). Non-linear developmental trajectory of electrical phenotype in rat substantia nigra pars compacta dopaminergic neurons. *eLife*, 3:e04059.

37. Price, K. V., Storn, R. M., and Lampinen, J. A. (2005). *Differential Evolution*. Springer-Verlag, Berlin Heidelberg.

38. Van Geit, W., Gevaert, M., Chindemi, G., Rössert, C., Courcol, J.-D., Muller, E. B., Schürmann, F., Segev, I., and Markram, H. (2016). Bluepyopt: Leveraging open source software and cloud infrastructure to optimise model parameters in neuroscience. *Frontiers in Neuroinformatics*, 10(17).

39. Bell, A. J. and Sejnowski, T. J. (1995). An information-maximisation approach to blind separation and blind deconvolution. *Neural Computation*, 7(6):1004–1034.

40. Octeau, J. C., Gangwani, M., Allam, S. L., Tran, D., Huang, S., Hoang Trong, T. M., Golshani, P., Rumbell, T. H., Kozloski, J. R., and Khakh, B. S. (2019). Transient, consequential increases in extracellular potassium ions accompany Channelrhodopsin2 (ChR2) excitation. *Cell Reports*, 27(8):2249–2261.E7.

41. Blythe, S. N., Wokosin, D., Atherton, J. F., and Bevan, M. D. (2009). Cellular mechanisms underlying burst firing in substantis nigra dopamine neurons. *The Journal of Neuroscience*, 29(49):15531–15541.

42. Häusser, M., Stuart, G., Racca, C. and Sakmann, B. (1995). Axonal initiation and active dendritic propagation of action potentials in substantia nigra neurons. *Neuron*, 15:637–647.

43. Carnevale, N. and Hines, M. (2006). *The NEURON Book*. Cambridge Univ. Press, New York.

44. Svensson, C.-M., Coombes, S., and Peirce, J. W. (2012). Using evolutionary algorithms for fitting high-dimensional models to neuronal data. *Neuroinformatics*, 10(2):199–218.

45. Vanier, M., and Bower J. A. (1999). A comparative survey of automated parameter-search methods for compartmental neural models. *Journal of Computational Neuroscience*, 7:149–171.

46. Van Geit, Achard, P., and de Schutter, E. (2008). Automated neuron model optimization techniques: a review. *Biological Cybernetics*, 99:241-251.

47. Achard, P., and de Schutter, E. (2006). Complex parameter landscape for a complex neuron model. *PLoS Computational Biology*, 2(7):e94.

48. Bahl, A., Stemmler, M. B., Herz, A. V. M., and Roth, A. (2012). Automated optimization of a reduced layer 5 pyramidal cell model based on experimental data. *Journal of Neuroscience Methods*, 210(1):22–34.

49. Hendrickson, E. B., Edgerton, J. R., and Jaeger, D. (2011). The use of automated parameter searches to improve ion channel kinetics for neural modeling. *Journal of Computational Neuroscience*, 31(2):329–46.

50. Rumbell, T. H., Draguljić, D., Yadav, A., Hof, P. R., Luebke, J. I., and Weaver, C. M. (2016). Automated evolutionary optimization of ion channel conductances and kinetics in models of young and aged rhesus monkey pyramidal neurons. *Journal of Computational Neuroscience*, 41(1):65–90.

51. Druckmann, S., Banitt, Y., Gidon, A., Schürmann, F., and Markram, H. (2007). A novel multiple objective optimization framework for constraining conductance-based neuron models. *Frontiers in Neuroscience*, 1(1):7–18.

52. Hay, E., Hill, S., Schürmann, F., Markram, H., and Segev, I. (2011). Models of neocortical layer 5b pyramidal cells capturing a wide range of dendritic and perisomatic active properties. *PLoS Computational Biology*, 7(7):e1002107.

53. Jędrzejewski-Szmek, Z., Abrahao, K. P., Jędrzejewski-Szmek, J., Lovinger, D., and Blackwell, K. T. (2018). Parameter optimization using covariance matrix adaptive-evolutionary strategy (CMA-ES), an approach to investigate differences in channel properties between neuron subtypes. *Frontiers in Neuroinformatics*, 12(47):1–20.

54. Masoli, S. Rizza, M. F., Sgritta, M., van Geit, W., Schürmann, F., and D'Angelo, E. (2017). Single neuron optimization as a basis for accurate biophysical modeling: the case of cerebellar granule cells. *Frontiers in Cellular Neuroscience*, 11:71.

55. Neymotin, S. A., Suter, B. A., Dura-Bernal, S., Shepherd, G. M. G., Migliore, M., and Lytton, W. W. (2017). Optimizing computer models of corticospinal neurons to replicate in vitro dynamics. *Journal of Neurophysiology*, 117:148–162.

56. Gouwens, N. W., Berg, J., Feng, D., Sorenson, S. A., Zeng, H., Hawrylycz, M. J., Koch, C., and Arkhipov, A. (2018). Systematic generation of biophysically detailed models for diverse cortical neuron types. *Nature Communications*, 9:710.

57. Ping, H. X. and Shepard, P. D. (1996). Apamin-sensitive Ca^{2+}-activated K^{+} channels regulate pacemaker activity in nigral dopamine neurons. *NeuroReport*, 7:809–814.

58. Devenyi, R. A. and Sobie, E. (2015). There and back again: iterating between population-based modeling and experiments reveals surprising regulation of calcium transients in rat cardiac myocytes. *Journal of Molecular and Cellular Cardiology*, 96:38–48.

59. Sarkar, A. X., Christini, D. J., and Sobie, E. A. (2012). Exploiting mathematical models to illuminate electrophysiological variability between individuals. *The Journal of Physiology*, 590(11):2555–2567.

60. Sarkar, A. X. and Sobie, E. A. (2010). Regression analysis for constraining free parameters in electrophysiological models of cardiac cells. *PLoS Computational Biology*, 6(9).

61. Drion, G., O'Leary, T., and Marder. E. (2015). Ion channel degeneracy enables robust and tunable neuronal firing rates. *PNAS*, 112(38):E5361–E5370.

62. Grace, A. A., and Bunney, B. S. (1984). The control of firing pattern in nigral dopamine neurons: burst firing. *The Journal of Neuroscience*, 4(11):2877–2890.

63. Ji, H., Hougaard, C., Herrik, K. F., Strøbæk, D., Christophersen, P., and Shepard, P. D. (2009). Tuning the excitability of midbrain dopamine neurons by modulating the Ca^{2+} sensitivity of SK channels. *European Journal of Neuroscience*, 29:1883–1895.

64. Kimm, T., Khaliq, Z. M. and Bean, B. P. (2015). Differential regulation of action potential shape and burst-frequency firing by BK and Kv2 channels in substantia nigra dopaminergic neurons. *The Journal of Neuroscience*, 35(50):16404–16417.

65. Grace, A. A., and Bunney, B. S. (1984). The control of firing pattern in nigral dopamine neurons: single spike firing. *The Journal of Neuroscience*, 4(11):2866–2876.

66. Grace, A. A. and Onn, S. P. (1989). Morphology and electrophysiological properties of immunocytochemically identified rat dopamine neurons recorded *in vitro*. *The Journal of Neuroscience*, 9(10):3463–3481.

67. Kozloski, J. (2016). Closed-loop brain model of neocortical information-based exchange. *Frontiers in Neuroanatomy*, 10:3.

68. Chan, C. S., Guzman, J. N., Ilijic, E., Mercer, J. N., Rick, C., Tkatch, T., Meredith, G. E., and Surmeier, D. J. (2007). 'Rejuvenation' protects neurons in mouse models of Parkinson's disease. *Nature*, 447(7148):1081–1086.

69. Putzier, I., Kullmann, P. H. M., Horn, J. P., and Levitan, E. S. (2009). $Ca_v1.3$ channel voltage dependence, not Ca^{2+} selectivity, drives pacemaker activity and amplifies bursts in nigral dopamine neurons. *The Journal of Neuroscience*, 29(49):15414–15419.

70. Canavier, C. C., Evans, R. K., Oster, A. M., Pissadaki, E. K., Drion, G., Kuznetsov, A. S., and Gutkin, B. S. (2016). Implications of cellular models of dopamine neurons for disease. *Journal of Neurophysiology*, 116:2815–2830.

71. Waroux, O., Massotte, L., Alleva, L., Graulich, A., Thomas, E., Liégeois, J.-F., Scuvée-Moreau, J., and Seutin, V. (2005). SK channels control the firing pattern of midbrain dopaminergic neurons *in vivo*. *European Journal of Neuroscience*, 22:3111–3121.

72. Qian, K., Yu, N., Tucker, K. R., Levitan, E. S., and Canavier, C. C. (2014). Mathematical analysis of depolarization block mediated by slow inactivation of fast sodium channels in midbrain dopamine neurons. *Journal of Neurophysiology*, 112:2779–2790.

73. Yu, N. and Canavier, C. C. (2015). A mathematical model of a midbrain dopamine neuron identifies two slow variables likely responsible for bursts evoked

by SK channel antagonists and terminated by depolarization block. *Journal of Mathematical Neuroscience*, 5(5):1–19.

74. Hay, E., Schürmann, F., Markram, H., and Segev, I. (2013). Preserving axosomatic spiking features despite diverse dendritic morphology. *Journal of Neurophysiology*, 109(12):2972–81.

75. Günay, C., Edgerton, J. R., and Jaeger, D. (2008). Channel density distributions explain spiking variability in the globus pallidus: a combined physiology and computer simulation database approach. *The Journal of Neuroscience*, 28(30):7476–91.

76. Morotti, S. and Grandi, E. (2017). Logistic regression analysis of populations of electrophysiological models to assess proarrythmic risk. *MethodsX*, 4:25–34.

77. Ni, H., Morotti, S., and Grandi, E. (2018). A heart for diversity: simulating variability in cardiac arrhythmia research. *Frontiers in Physiology*, 9:958.

78. Lawson, B. A. J., Drovandi, C. C., Cusimano, N., Burrage, P., Rodriguez, B., and Burrage, K. (2018). Unlocking data sets by calibrating populations of models to data density: a study in atrial electrophysiology. *Science Advances*, 4:e1701676.

79. O'Leary, T., Williams, A. H., Franci, A., and Marder, E. (2014). Cell types, network homeostasis, and pathological compensation from a biologically plausible ion channel expression model. *Neuron*, 82(4):809–821.

80. Ransdell, J. L., Nair, S. S., and Schulz, D. J. (2013). Neurons within the same network independently achieve conserved output by differentially balancing variable conductance magnitudes. *Journal of Neuroscience*, 33(24):9950–9956.

81. Canavier, C. C., Oprisan, S. A., Callaway, J. C., Ji, H., and Shepard, P. D. (2007). Computational model predicts a role for ERG current in repolarizing plateau potentials in dopamine neurons: implications for modulation of neuronal activity. *Journal of Neurophysiology*, 98(5):3006–3022.

82. Kang, Y. and Kitai, S. T. (1993a). A whole cell patch-clamp study on the pacemaker potential in dopaminergic neurons of rat substantia nigra compacta. *Neuroscience Research*, 18:209–221.

83. Memelli, H., Torben-Nielsen, B., Kozloski, J. (2013). Self-referential forces are sufficient to explain different dendritic morphologies. *Frontiers in Neuroinformatics*, 7:1.

84. Tucker, K. R., Huertas, M. A., Horn, J. P., Canavier, C. C., and Levitan, E. S. (2012). Pacemaker rate and depolarization block in nigral dopaminergic neurons: a somatic sodium channel balancing act. *The Journal of Neuroscience*, 32(42):14519-14531.

85. Seutin, V. and Engel, D. (2010). Differences in Na^+ channel functional properties between dopamine and GABA neurons of the rat substantia nigra. *Journal of Neurophysiology*, 103:3099–3114.

86. Ding, S., Wei, W., and Zhou, F. -M. (2011). Molecular and functional differences in voltage-activated sodium currents between GABA projection neurons and dopamine neurons in the substantia nigra. *Journal of Neurophysiology*, 106:3019–3034.

87. Mercuri, N. B., Calabresi, P., Stefani, A., and Bernardi, G. (1995). Properties of the hyperpolarization-activated cation current I_h in rat midbrain dopaminergic neurons. *European Journal of Neuroscience*, 7:462–469.

88. Wang, J., Chen, S., Nolan, M. F., and Siegelbaum, S. A. (2002). Activity-dependent regulation of HCN pacemaker channels by cyclic AMP: signaling through dynamic allosteric coupling. *Neuron*, 36(3):451–461.

89. Pissadaki, E. K. and Bolam, J. P. (2013). The energy cost of action potential propagation in dopamine neurons: clues to susceptibility in Parkinson's disease. *Frontiers in Computational Neuroscience*, 7(March):1–17.

90. Gambardella, C., Pignatelli, A., and Belluzzi, O. (2012). The h-current in the substantia nigra pars compacta neurons: a re-examination. *PLoS ONE*, 7(12).

91. McRory, J. E., Santi, C. M., Hamming, K. S. C., Mezeyova, J., Sutton, K. G., Baillie, D. L., Stea, A., and Snutch, T. P. (2001). Molecular and functional characterization of a family of rat brain T-type calcium channels. *The Journal of Biological Chemistry*, 276(6):3999–4011.

92. Poetschke, C., Dragicevic, E., Duda, J., Benkert, J., Dougalis, A., Dezio, R., Snutch, T. P., Striessnig, J., and Liss, B. (2015). Compensatory T-type Ca^{2+} channel activity alters D2-autoreceptor responses of Substantia nigra dopamine

neurons from Cav1.3 L-type Ca^{2+} channel KO mice. *Scientific Reports*, 5(13688):1–16.

93. Wolfart, J. and Roeper, J. (2002). Selective coupling of T-type calcium channels to SK potassium channels prevents intrinsic bursting in dopaminergic midbrain neurons. *Journal of Neuroscience*, 22(9):3404–3413.

94. Cui, G., Okamoto, T., and Morikawa, H. (2004). Spontaneous opening of T-type Ca^{2+} channels contributes to the irregular firing of dopamine neurons in neonatal rats. *The Journal of Neuroscience*, 24(49):11079–11087.

95. Evans, R. C. and Khaliq, Z. M. (2015). T-type calcium channels trigger a hyperpolarization induced afterdepolarization in substantia nigra dopamine neurons. *BMC Neuroscience*, 16(Suppl 1):P123.

96. Dufour, M. A., Woodhouse, A., and Goaillard, J.-M. (2014). Somatodendritic ion channel expression in substantia nigra pars compacta dopaminergic neurons across postnatal development. *Journal of Neuroscience Research*, 92:981–999.

97. Hering, J., Feltz, A., and Lamber, R. C. (2004). Slow inactivation of the $Ca_v3.1$ isotope of T-type calcium channels. *Journal of Physiology*, 555:331–344.

98. Durante, P., Cardenas, C. G., Whittaker, J. a., Kitai, S. T., and Scroggs, R. S. (2004). Low-threshold L-type calcium channels in rat dopamine neurons. *Journal of Neurophysiology*, 91(3):1450–1454.

99. Philippart, F., Destreel, G., Merino-Sepúlveda, P., Henny, P., Engel, D., and Seutin, V. (2016). Differential Somatic Ca^{2+} Channel Profile in Midbrain Dopaminergic Neurons. *The Journal of Neuroscience*, 36(27):7234–7245.

100. Koschak, A., Reimer, D., Huber, I., Grabner, M., Glossmann, H., Engel, J., and Striessnig, J. (2001). α1D (Cav1.3) subunits can form L-type Ca^{2+} channels activating at negative voltages. *The Journal of Biological Chemistry*, 276(25):22100–22106.

101. Moreno, C. M., Dixon, R. E., Tajada, S., Yuan, C., Opitz-Araya, X., Binder, M. D., and Santana, L. F. (2016). Ca^{2+} entry into neurons is facilitated by cooperative gating of clustered $Ca_v1.3$ channels. *eLife*, e15744:1–26.

102. Canavier, C. C. and Landry, R. S. (2006). An increase in AMPA and a decrease in SK conductance increase burst firing by different mechanisms in a model of a dopamine neuron in vivo. *Journal of Neurophysiology*, 96(5):2549–2563.

103. Komendantov, A. O., Komendantova, O. G., Johnson, S. W., and Canavier, C. C. (2004). A modeling study suggests complementary roles for $GABA_A$ and NMDA receptors and the SK channel in regulating the firing pattern in midbrain dopamine neurons. *Journal of Neurophysiology*, 91:346–357.

104. Migliore, M., Cannia, C., and Canavier, C. C. (2008). A modeling study suggesting a possible pharmacological target to mitigate the effects of ethanol on reward-related dopaminergic signaling. *Journal of Neurophysiology*, 99:2703–2707.

105. Kuznetsov, A. S., Kopell, N. J., Wilson, C. J., Alexey, S., Kopell, N. J., and Wilson, C. J. (2006). Transient high-frequency firing in a coupled-oscillator model of the mesencephalic dopaminergic neuron. *Journal of Neurophysiology*, 95:932–947.

106. Lipscombe, D., Helton, T. D., and Xu, W. (2004). L-Type calcium channels: the low down. *Journal of Neurophysiology*, 92:2633–2641.

107. Tuckwell, H. C. (2012). Quantitative aspects of L-type Ca^{2+} currents. *Progress in Neurobiology*, 96:1–31.

108. Helton, T. D., Xu, W., and Lipscombe, D. (2005). Neuronal L-type calcium channels open quickly and are inhibited slowly. *The Journal of Neuroscience*, 25(44):10247–10251.

109. Cui, G., Cox, D. H., and Aldrich, R. W. (1997). Intrinsic voltage dependence and Ca^{2+} regulation of *mslo* large conductance Ca-activated K^+ channels. *Journal of General Physiology*, 109:647–673.

110. Clay, J. R. (2017). Novel description of the large conductance Ca^{2+}-modulated K^+ channel current, BK, during an action potential from suprachiasmatic nucleus neurons. *Physiological Reports*, 5(20):e13473.

111. Tateno, T. (2010). A small-conductance Ca^{2+}-dependent K^+ current regulates dopamine neuron activity: a combined approach of dynamic current clamping and intracellular imaging of calcium signals. *NeuroReport*, 21(10):667–674.

112. Adelman, J. P., Maylie, J., and Sah, P. (2012). Small-conductance Ca^{2+}-activated K^+ channels: form and function. *Annual Review of Physiology*, 74:245–269.

113. Hirschberg, B., Maylie, J., Adelman, J. P., and Marrion, N. V. (1998). Gating of recombinant small-conductance Ca-activated K^+ channels by calcium. *The Journal of General Physiology*, 111(4):565–81.

114. Xia, X.-M., Fakler, B., Rivard, A., Wayman, G., Johnson-Pais, T., Keen, J. E., Ishii, T., Hirschberg, B., Bond, C. T., Lutsenko, S., Maylie, J., and Adelman, J. P. (1998). Mechanism of calcium gating in small-conductance calcium-activated potassium channels. *Nature*, 295:503–507.

115. Liss, B., Bruns, R., and Roeper, J. (1999). Alternative sulfonylurea receptor expression defines metabolic sensitivity of K-ATP channels in dopaminergic midbrain neurons. *The EMBO Journal*, 18(4):833–846.

116. Silva, N. L., Pechura, C. M., and Barker, J. L. (1990). Postnatal rat nigrostriatal dopaminergic neurons exhibit five types of potassium conductances. *Journal of Neurophysiology*, 64(1):262–272.

117. Liss, B. and Roeper, J. (2007). Individual dopamine midbrain neurons: functional diversity and flexibility in health and disease. *Brain Research Reviews*, 58:314–321.

118. Shepard, P. D. and Bunney, B. S. (1991). Repetitive firing properties of putative dopamine-containing neurons in vitro: regulation by an apamin-sensitive Ca^{2+}-activated K^+ conductance. *Experimental Brain Research*, 81(1):141–150.

119. Nedergaard, S. (2004). A Ca^{2+}-independent slow afterhyperpolarization in substantia nigra compacta neurons. *Neuroscience*, 125:841–852.

120. Ficker, E., Jarolimek, W., Kiehn, J., Baumann, A., and Brown, A. M. (1998). Molecular determinants of Dofetilide block of HERG K^+ channels. *Circulation Research*, 82(3):386–395.

121. Wang, S., Liu, S., Morales, M. J., Strauss, H. C., and Rasmusson, R. L. (1997). A quantitative analysis of the activation and inactivation kinetics of SHEROG expressed in Xenopus oocytes. *Journal of Physiology*, 502(1):45–60.
