# Supplementary figures and images for: Dimensions of control for subthreshold oscillations and spontaneous firing in dopamine neurons
Source: PLoS Comput Biol. 2019 Sep 23;15(9):e1007375. doi: 10.1371/journal.pcbi.1007375 (PMC6776370; doi:10.1371/journal.pcbi.1007375)

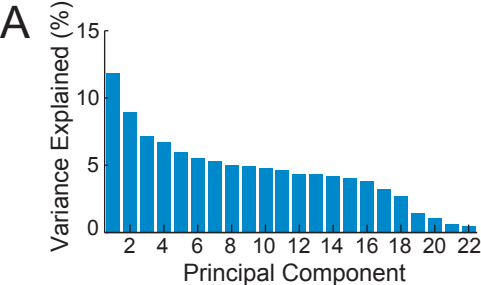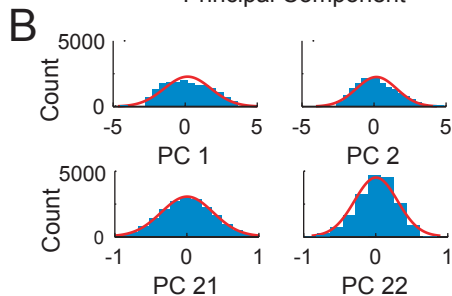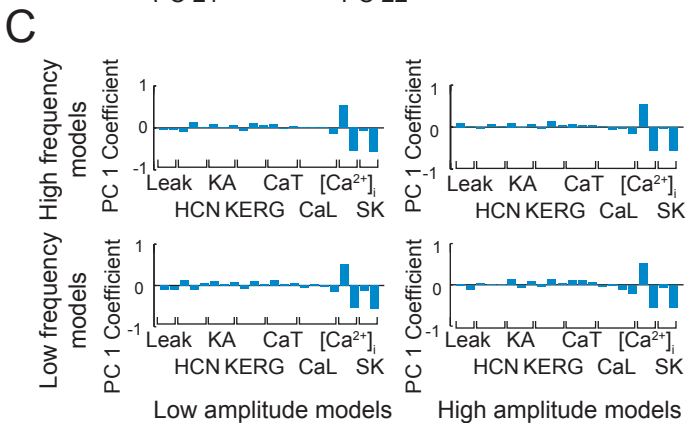

Supplement: S1 Fig — (A) PCA: variance explained (% total variance among parameter sets) by each PC. (B) Histogram of scores for the model population for 4 of the PCs. Red lines represent best fit Gaussian distribution centered at 0. (C) An axis of robustness in parameter space was discovered by performing PCA independently on nine populations of models, separated by dividing feature space into a uniform three by three grid according to STO frequency and amplitude. Bar plots show the first PC from independent PCA on parameters from the 4 corner groups of models. The major axis of variance through parameter space is always identical, regardless of location of model in feature space, indicating that parameters must be maintained according to this ratio for a model to be discovered as a ‘good’ model. (PDF) [file pcbi.1007375.s002.pdf]

**A**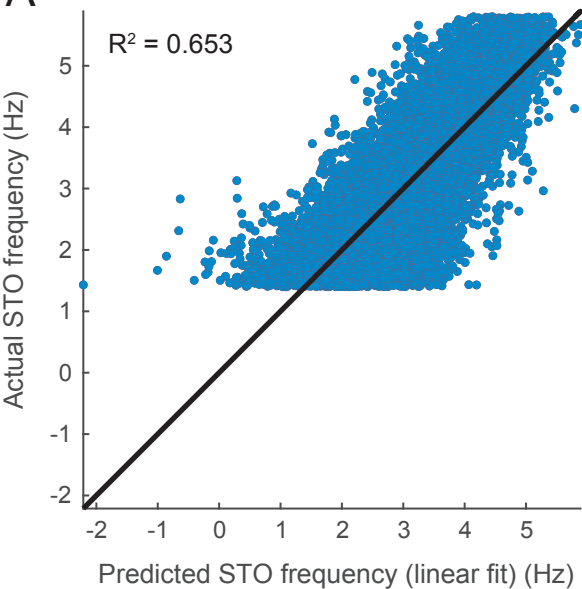**C**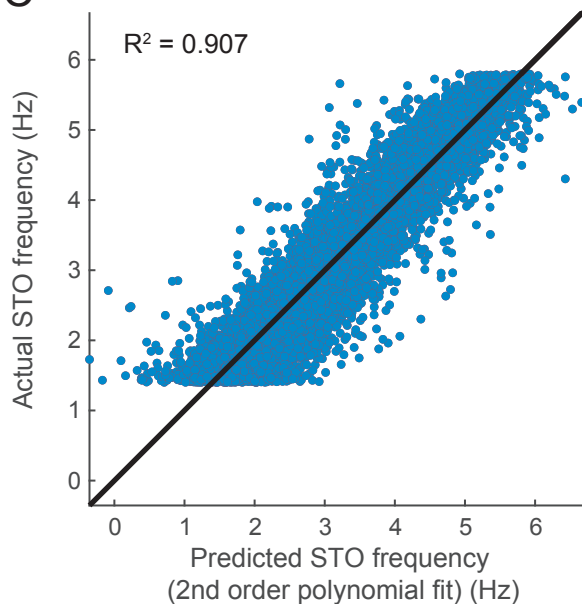**B**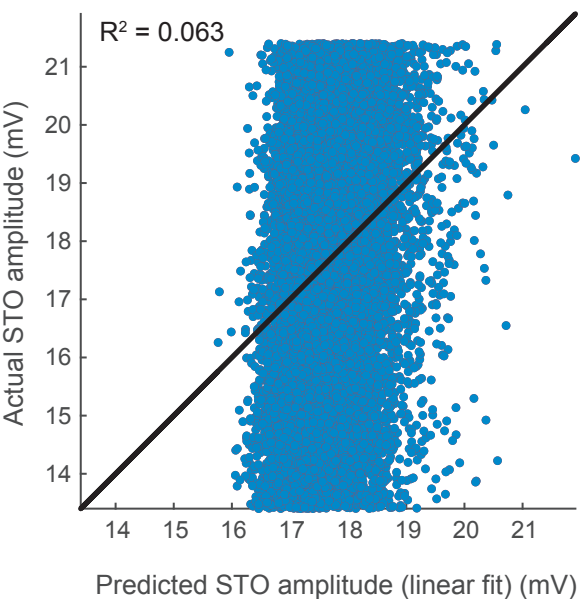**D**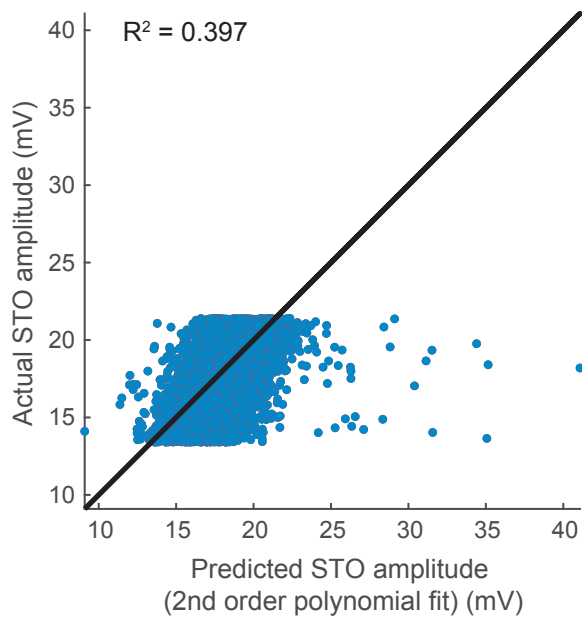

Supplement: S2 Fig — First-order and second-order fits of parameters to features using polynomial regression (Matlab package MultiPolyRegress (https://github.com/ahmetcecen/MultiPolyRegress-MatlabCentral)). Plots show actual feature value (y-axis) and feature value predicted by each parameter set according to the fix (x-axis). A) Linear fit to STO frequency. B) Linear fit to STO amplitude. C) Second order polynomial fit to STO frequency. D) Second order polynomial fit to STO amplitude. (PDF) [file pcbi.1007375.s003.pdf]

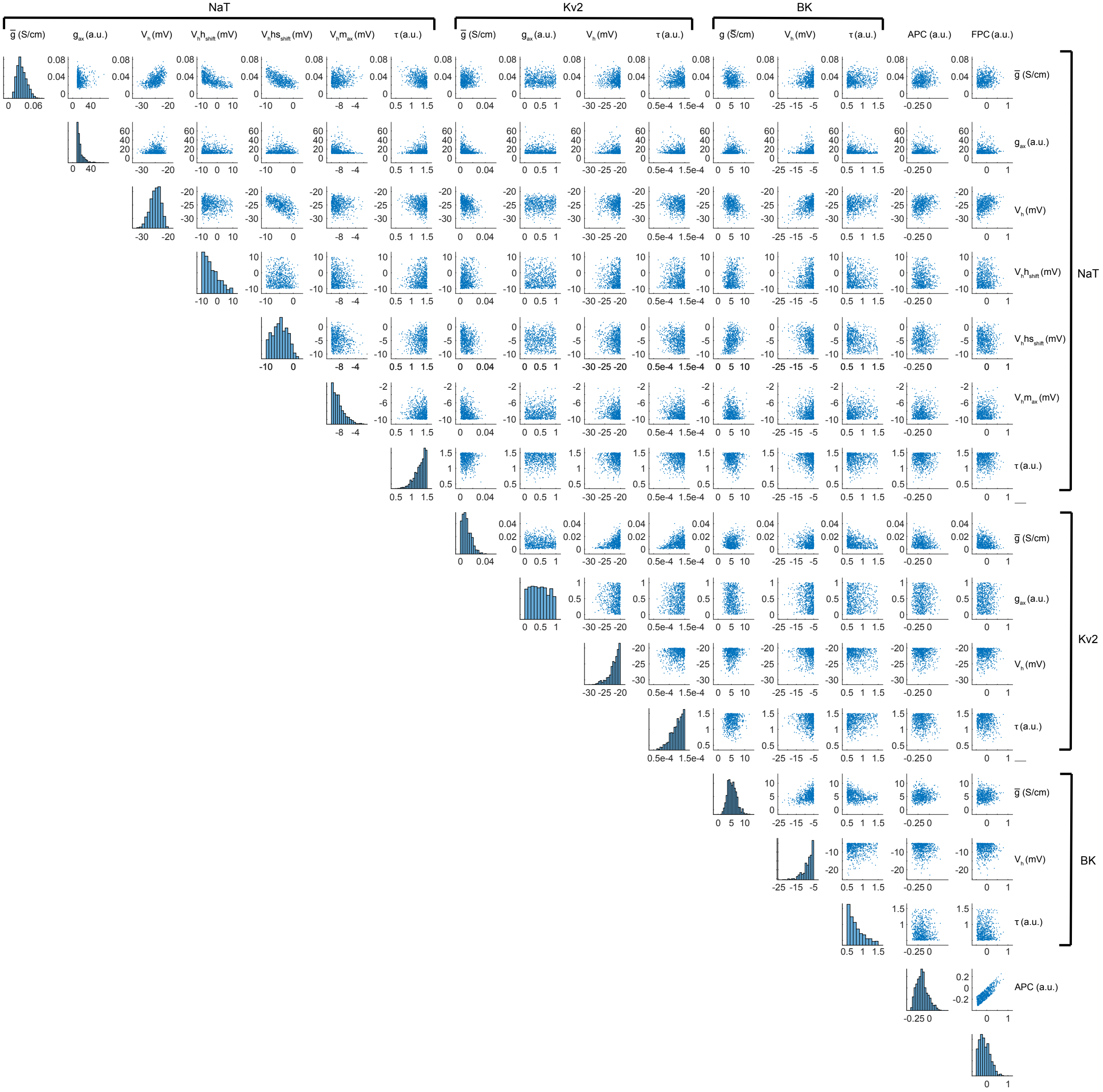

Supplement: S3 Fig — Histograms and pairwise comparison scatter plots of parameter values for all 727 good models from the final population of the spontaneous firing optimization. (PDF) [file pcbi.1007375.s004.pdf]

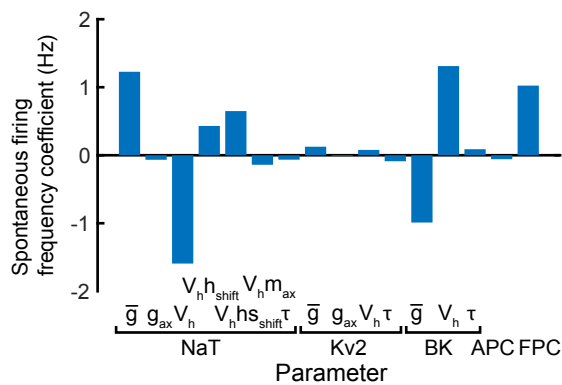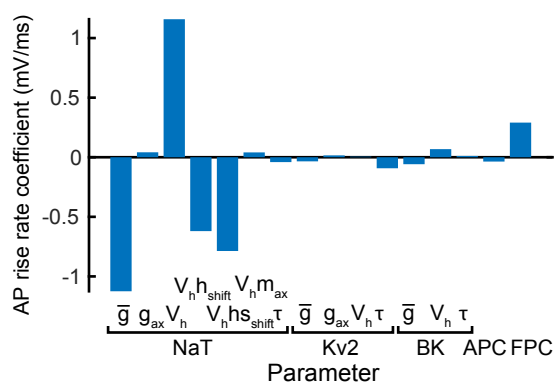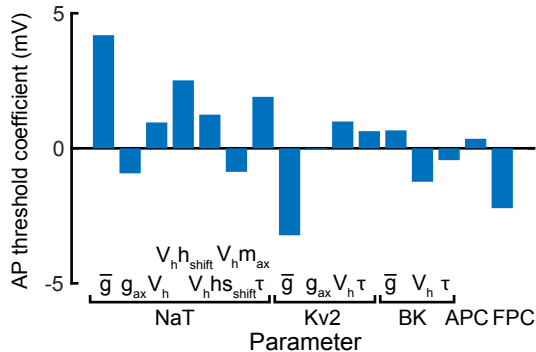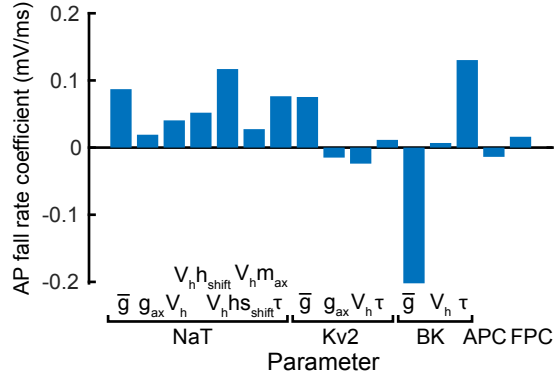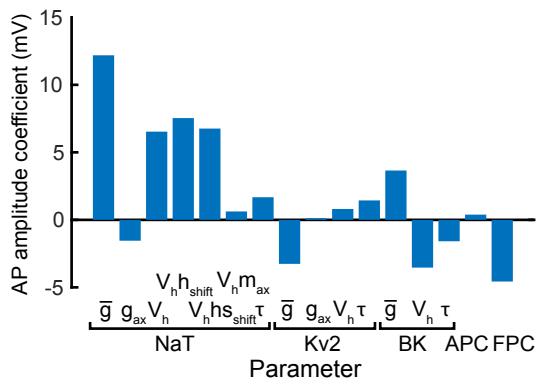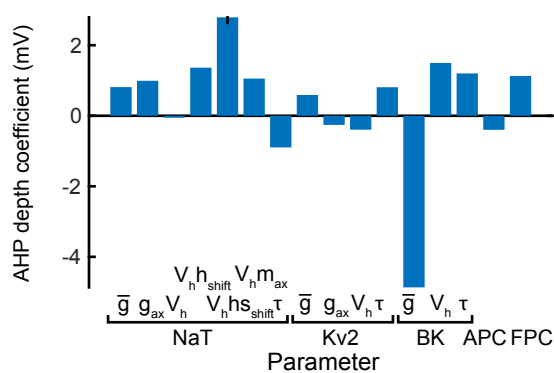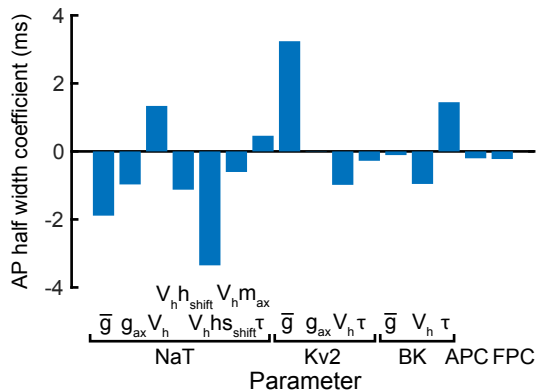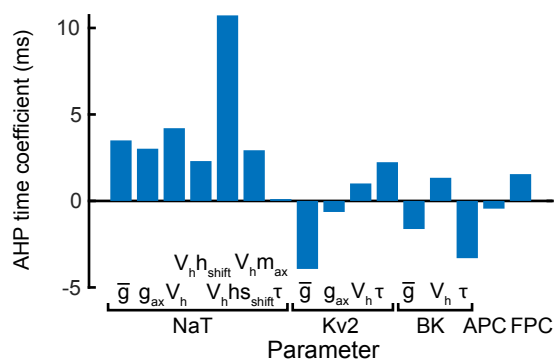

Supplement: S4 Fig — Feature-predicting coefficients from conducting PLSR on all 727 good models from the final population of the spontaneous firing optimization, for spiking features. (PDF) [file pcbi.1007375.s005.pdf]
