# Supplementary material for: Dimensions of control for subthreshold oscillations and spontaneous firing in dopamine neurons
Source: PLoS Comput Biol. 2019 Sep 23;15(9):e1007375. doi: 10.1371/journal.pcbi.1007375 (PMC6776370; doi:10.1371/journal.pcbi.1007375)
Supplement: S5 Fig — Schematic illustration of feature calculations to capture features listed in Table 1. (PDF) [file pcbi.1007375.s006.pdf]

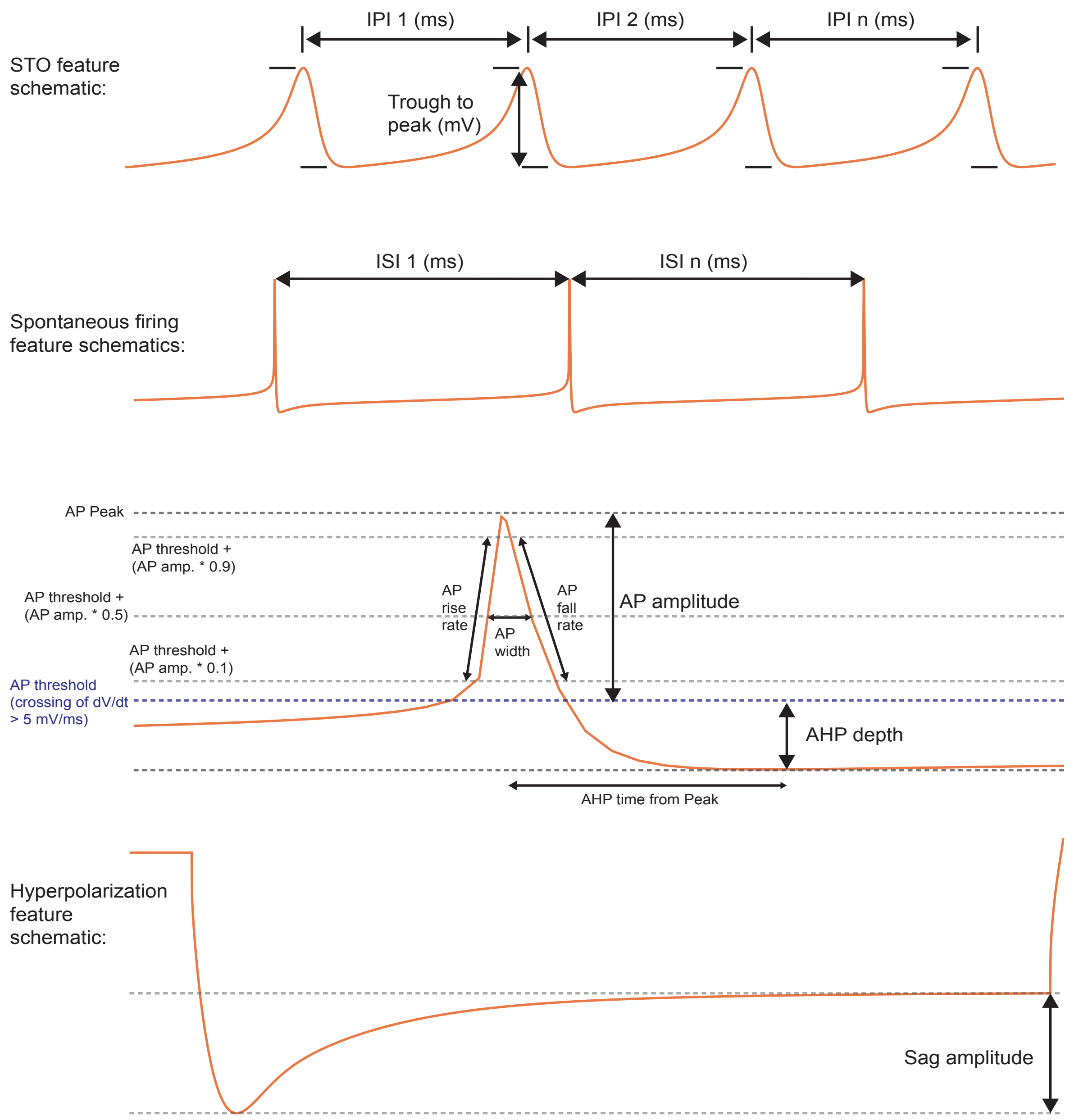

STO feature schematic:
IPI 1 (ms)
IPI 2 (ms)
IPI n (ms)
Trough to peak (mV)
Spontaneous firing feature schematics:
ISI 1 (ms)
ISI n (ms)
AP Peak
AP threshold + (AP amp. * 0.9)
AP threshold + (AP amp. * 0.5)
AP threshold + (AP amp. * 0.1)
AP threshold (crossing of dV/dt > 5 mV/ms)
AP rise rate
AP width
AP fall rate
AP amplitude
AHP depth
AHP time from Peak
Hyperpolarization feature schematic:
Sag amplitude
